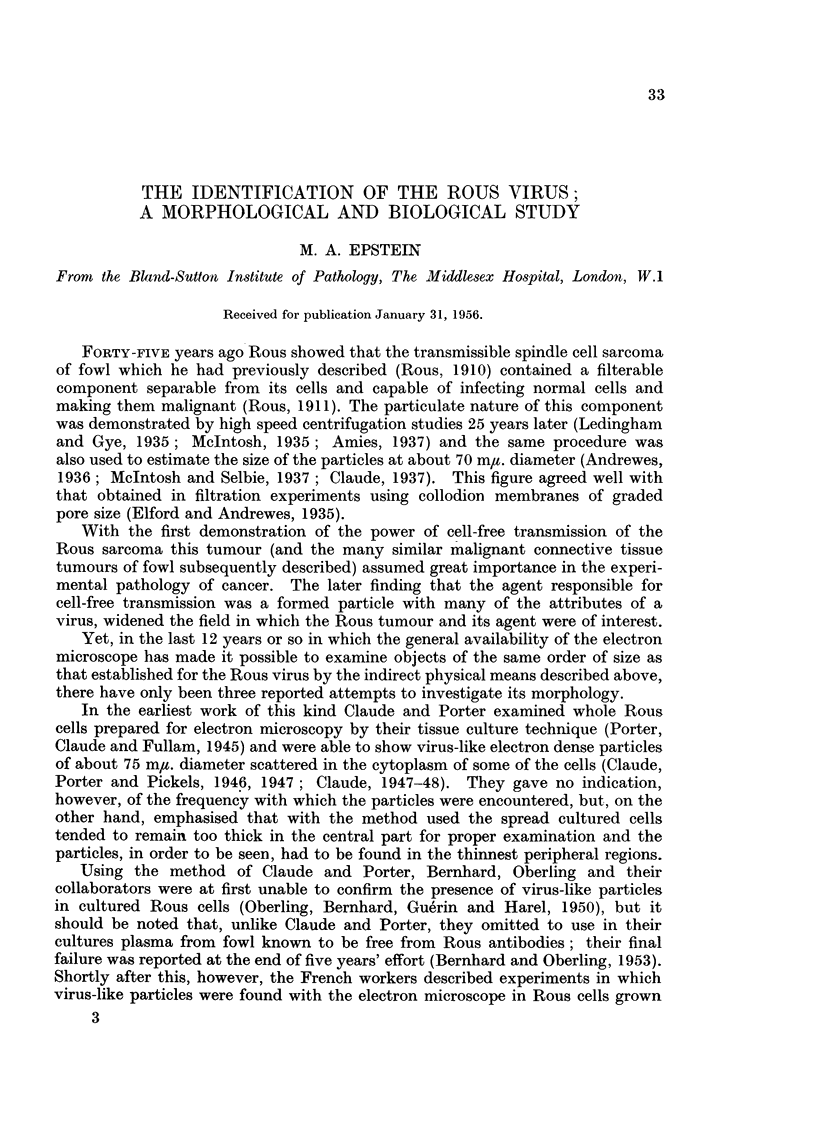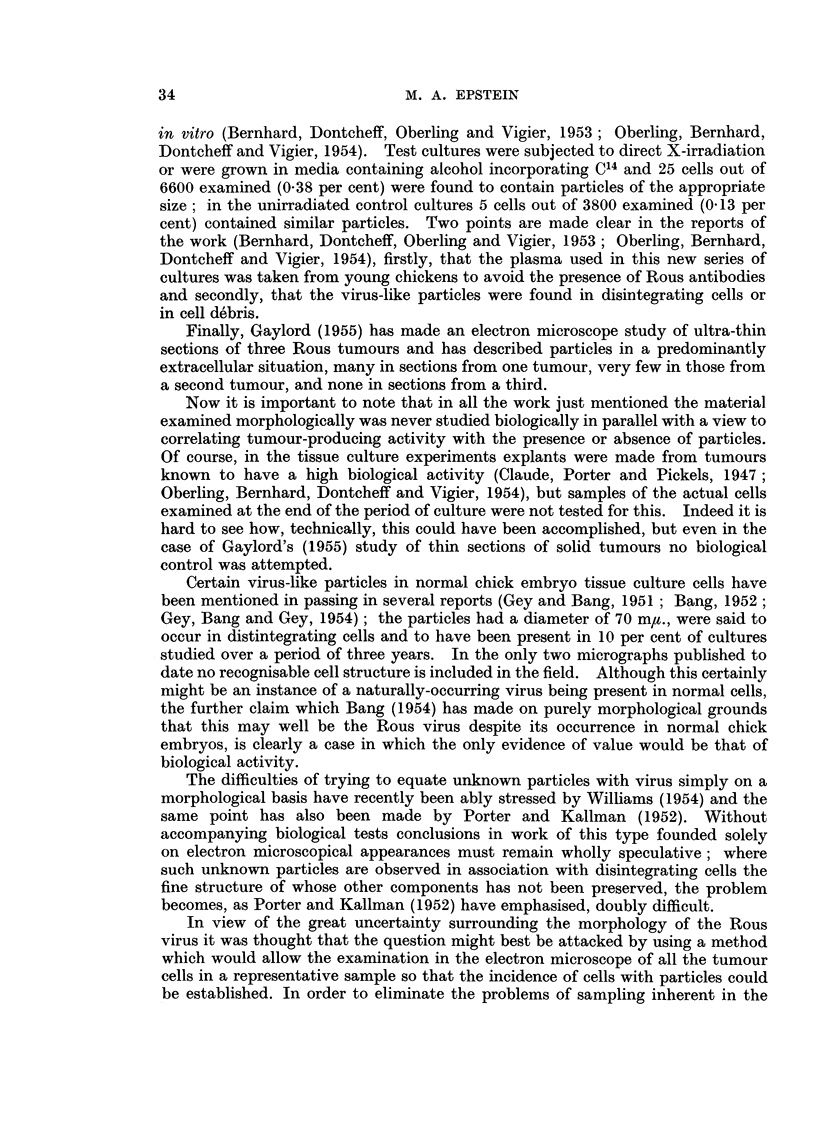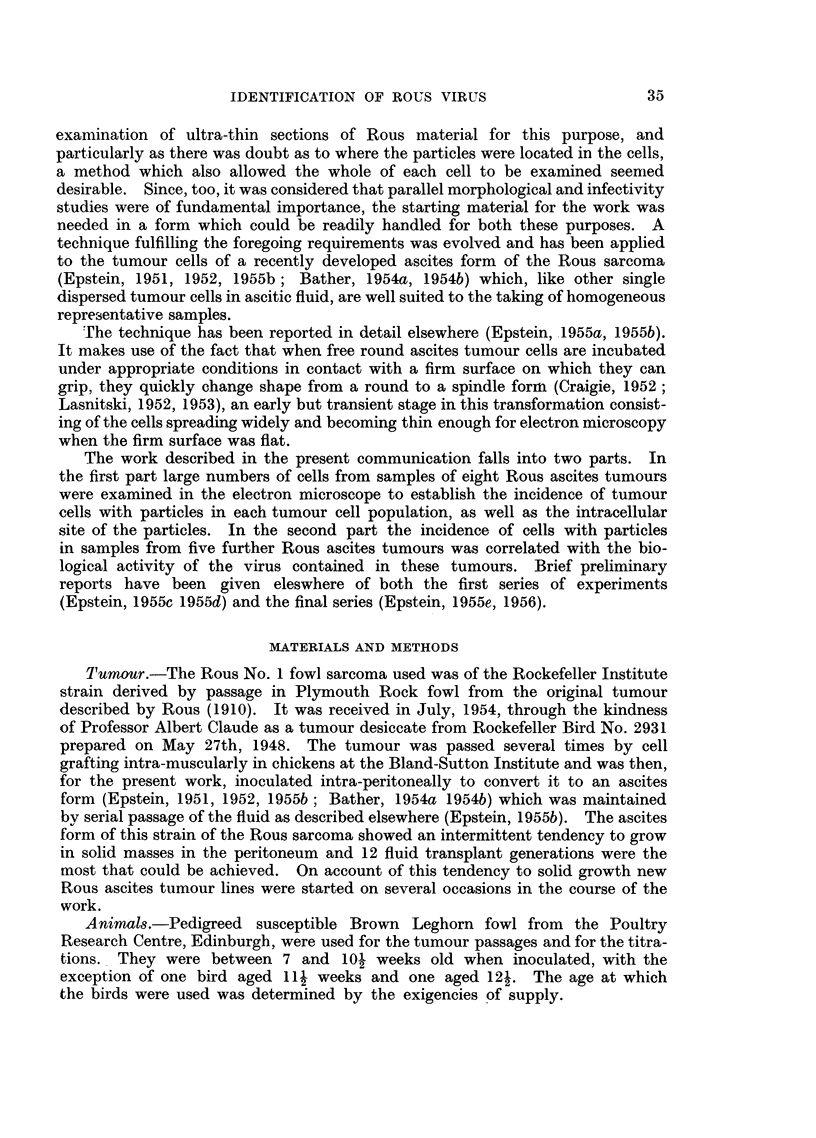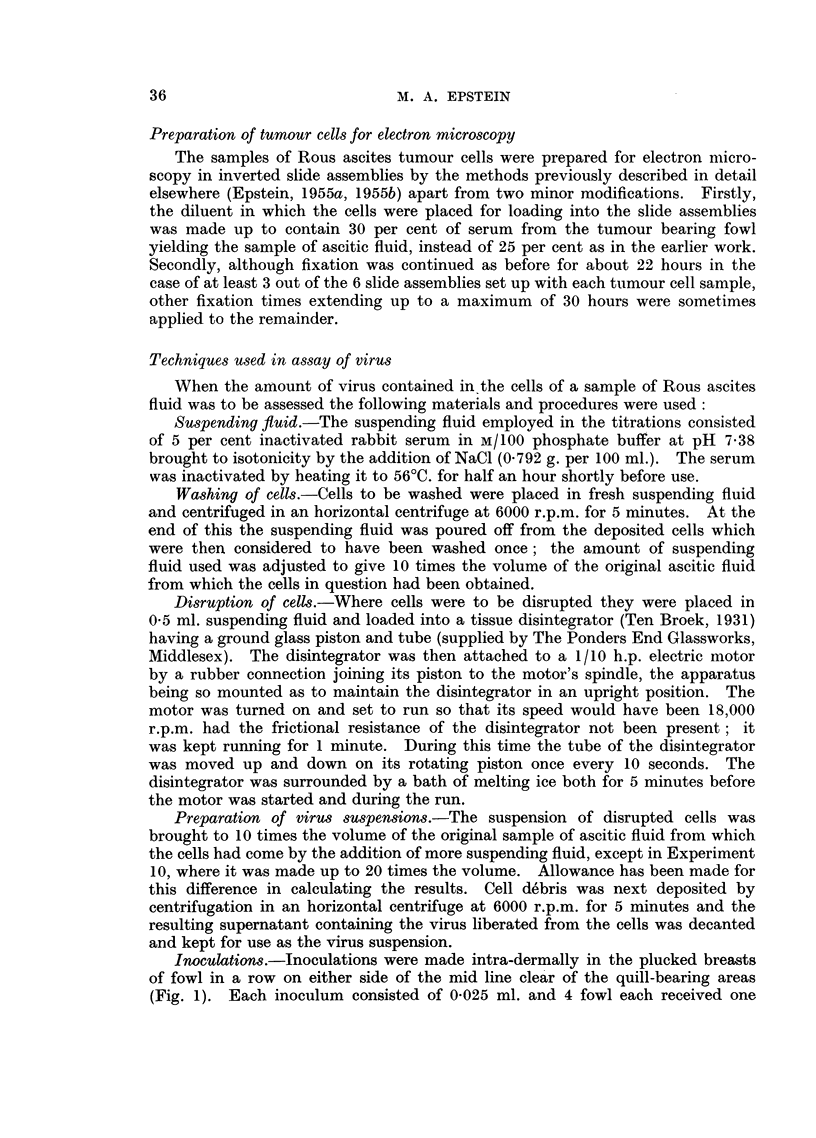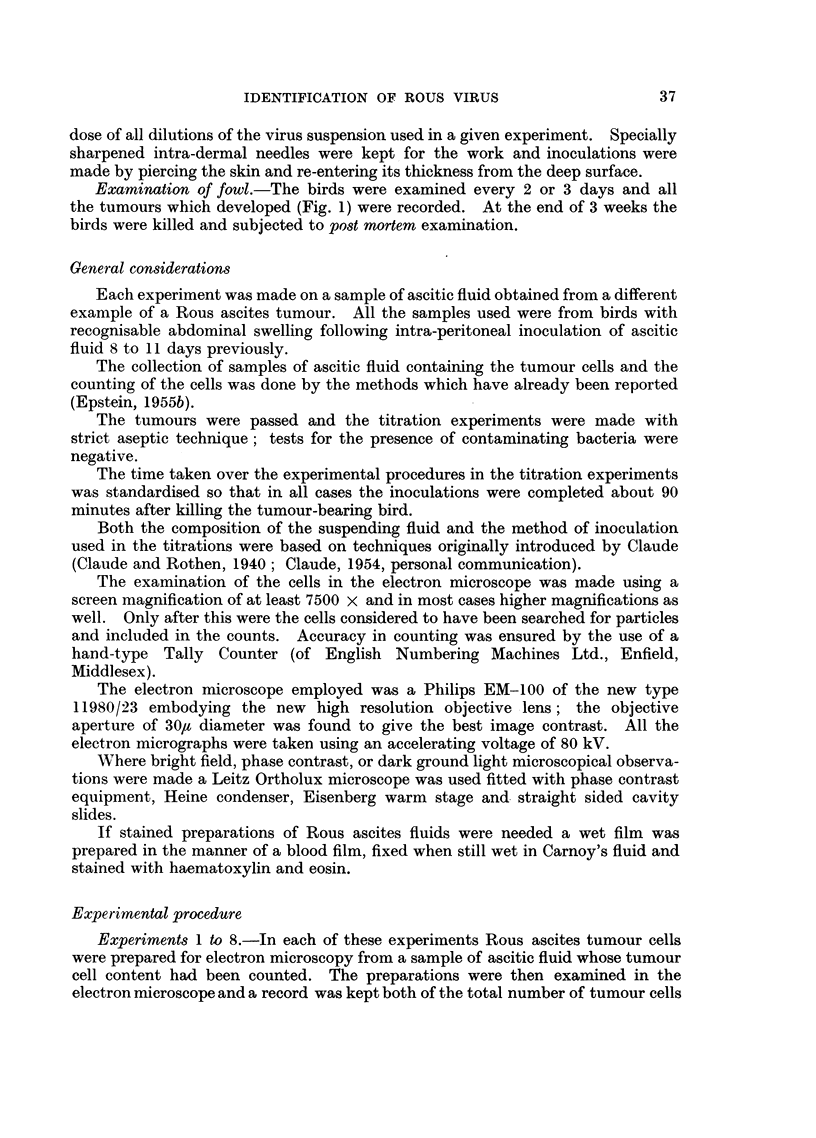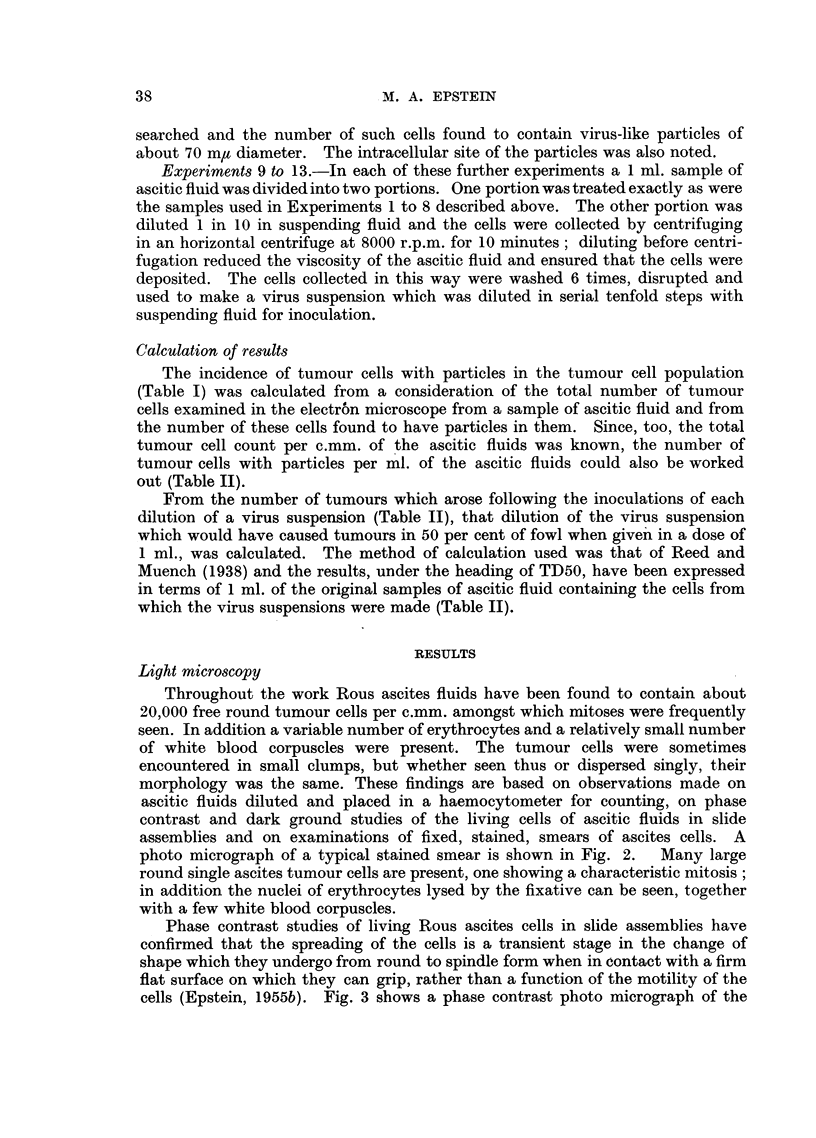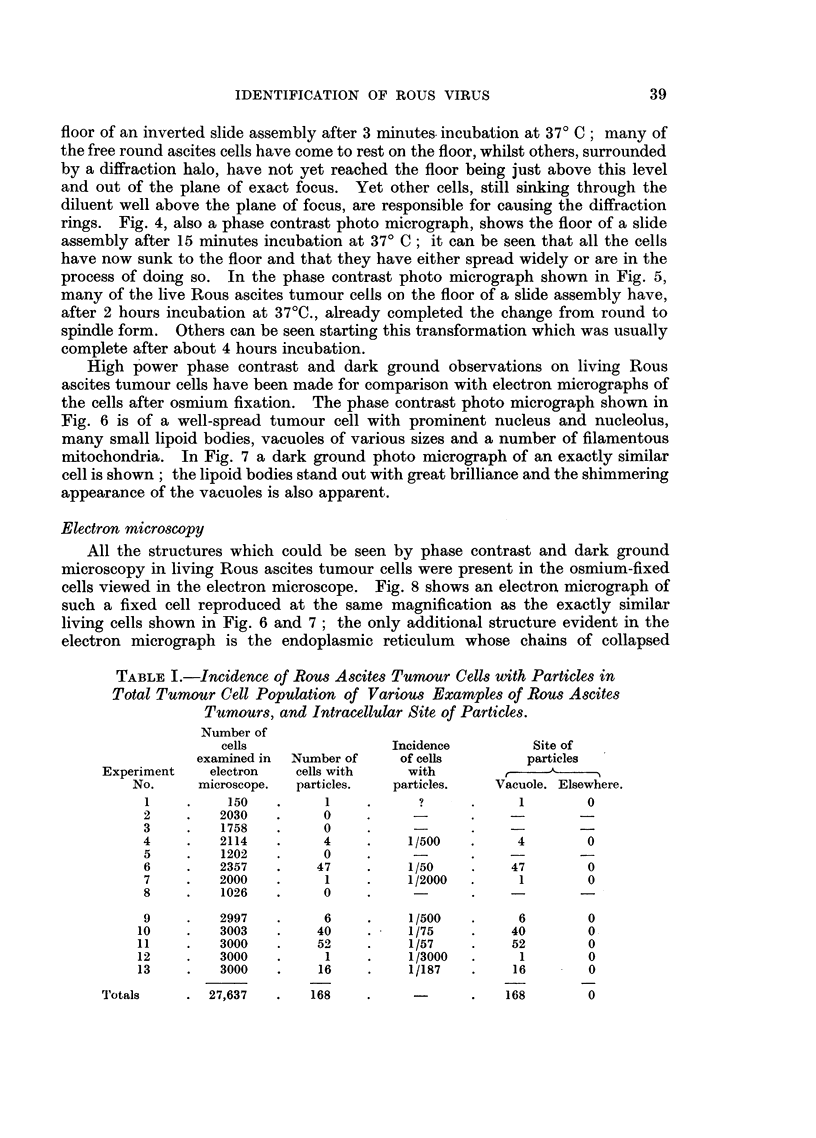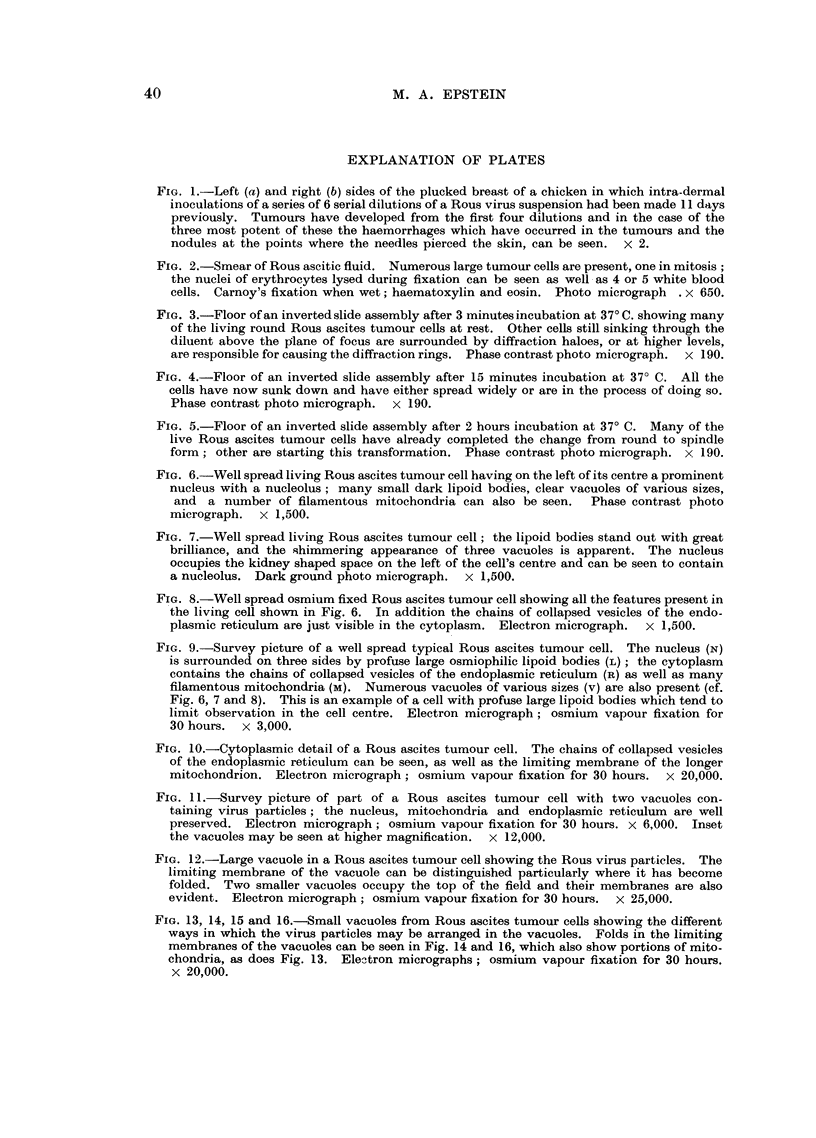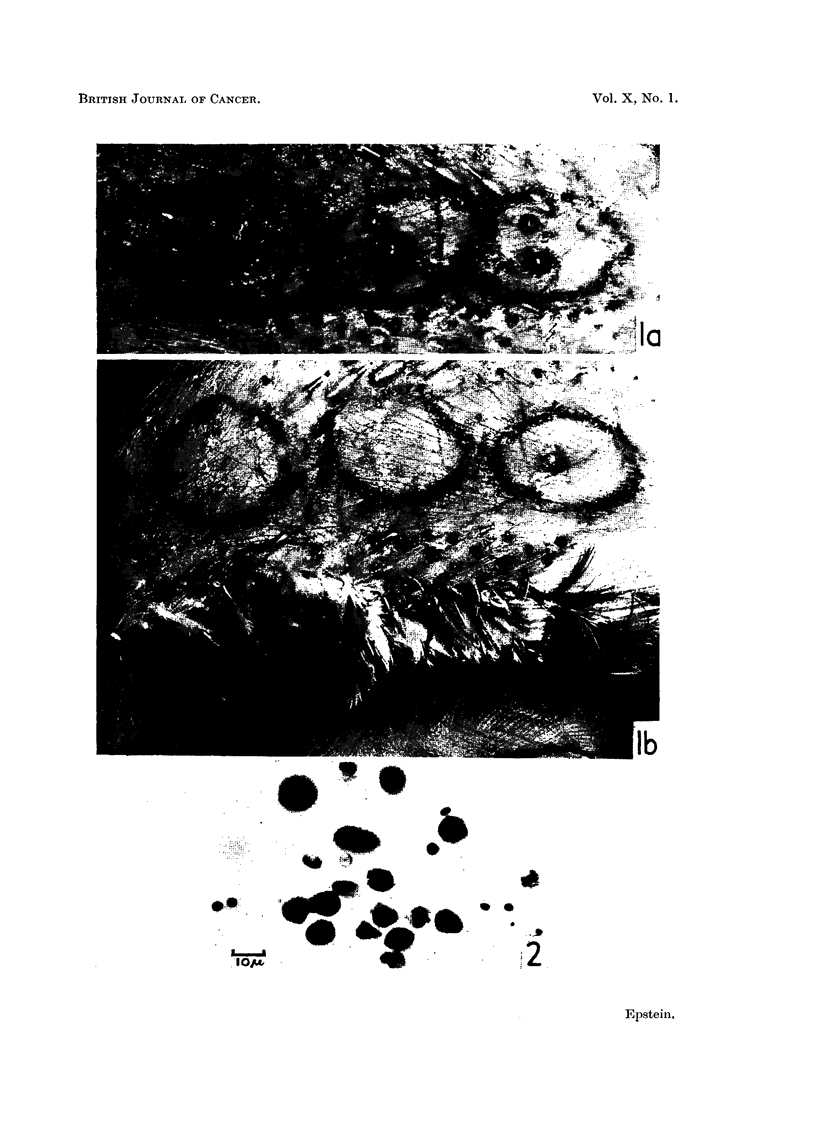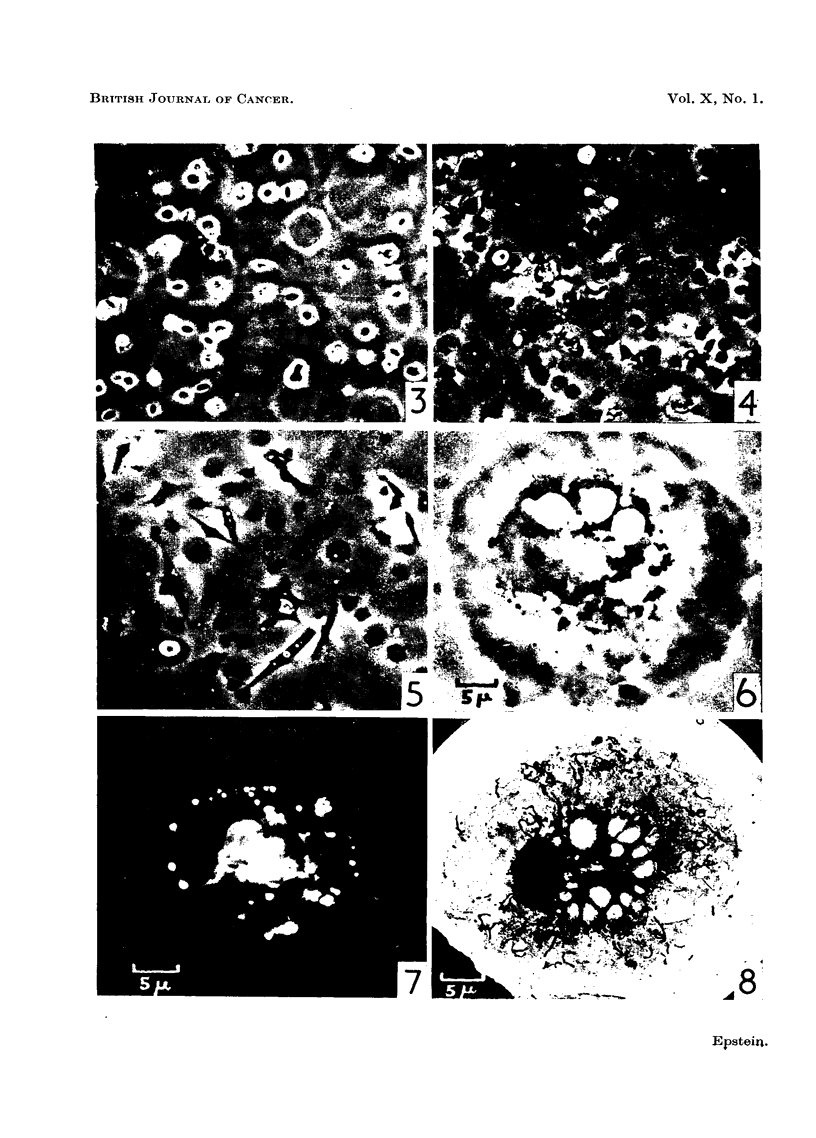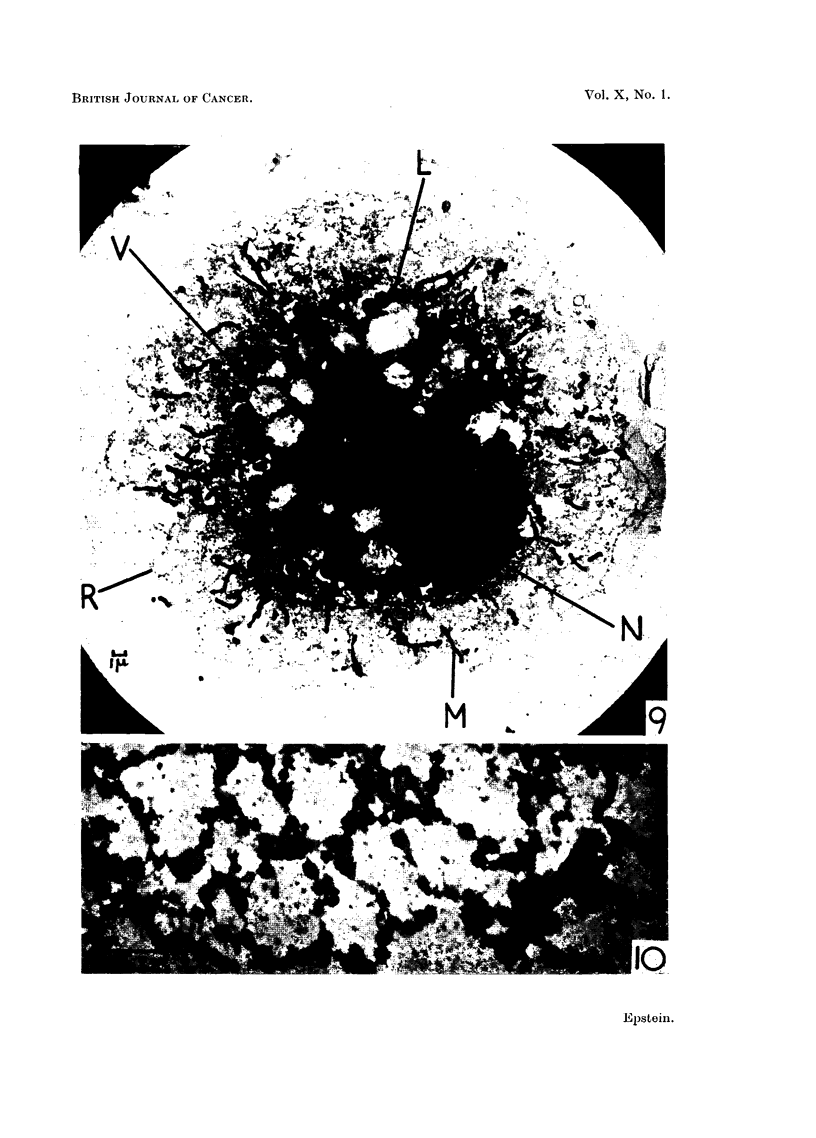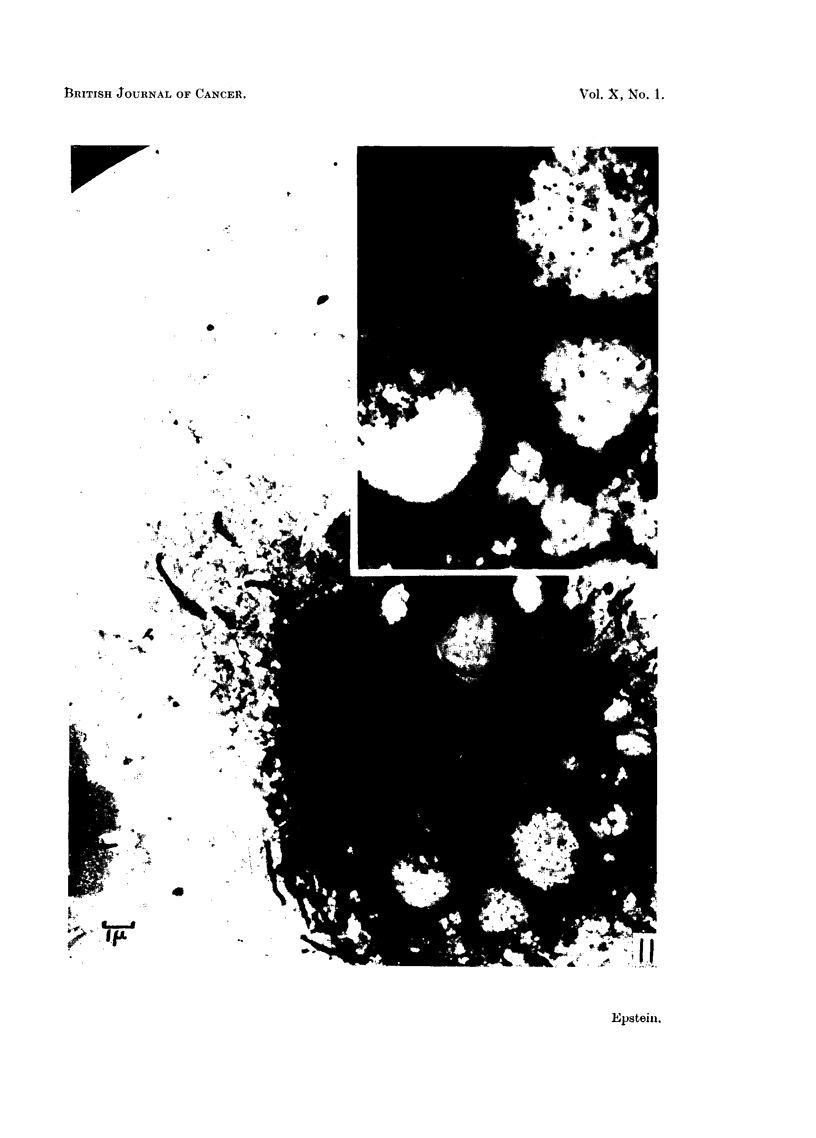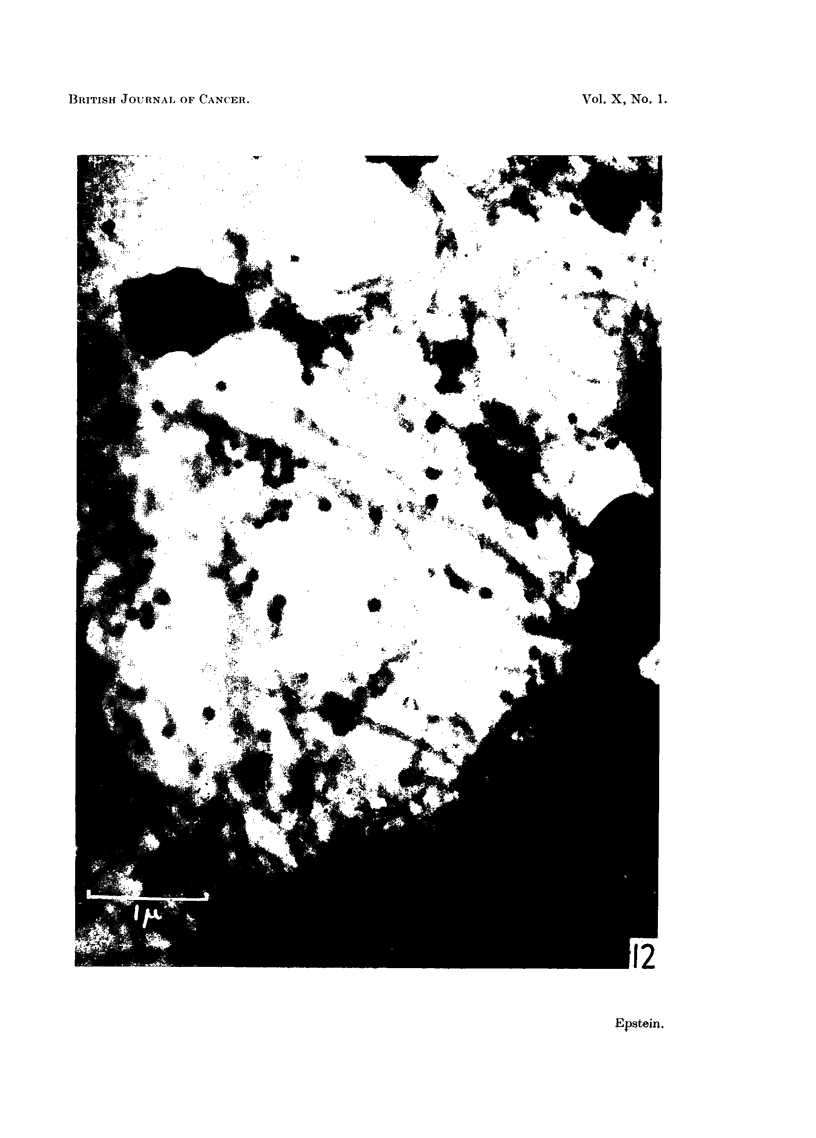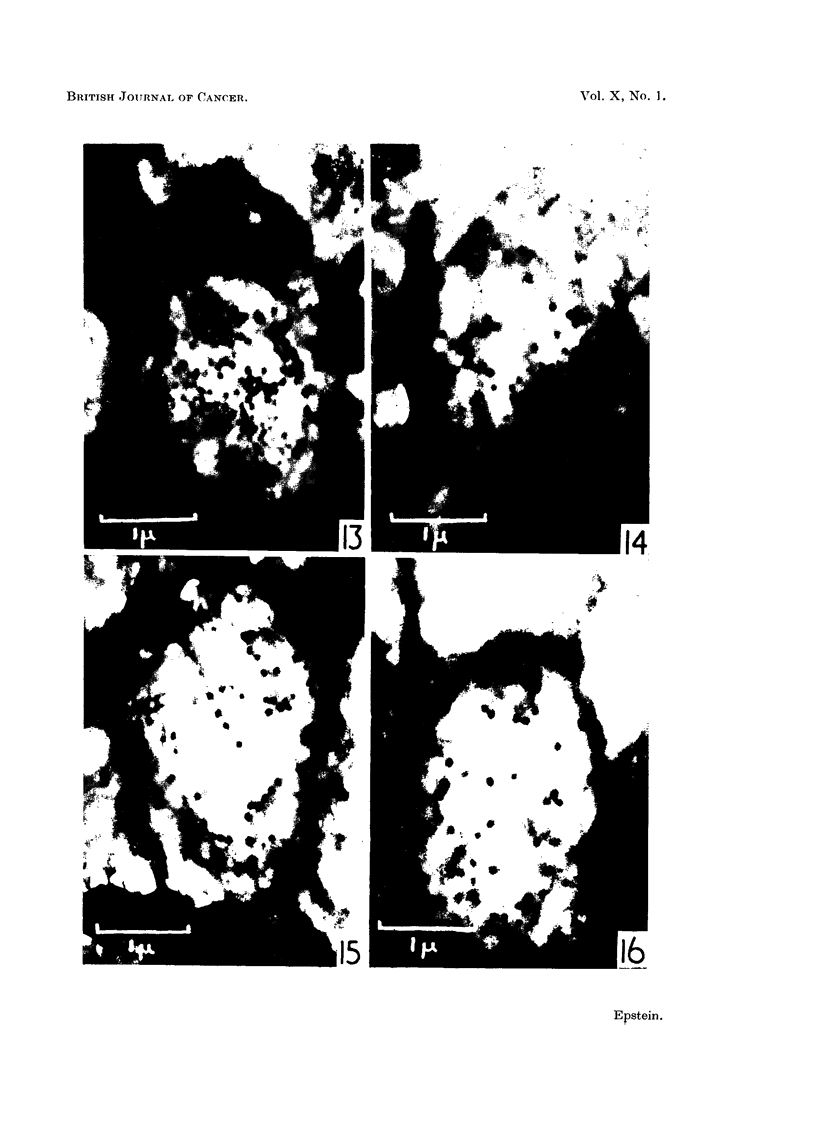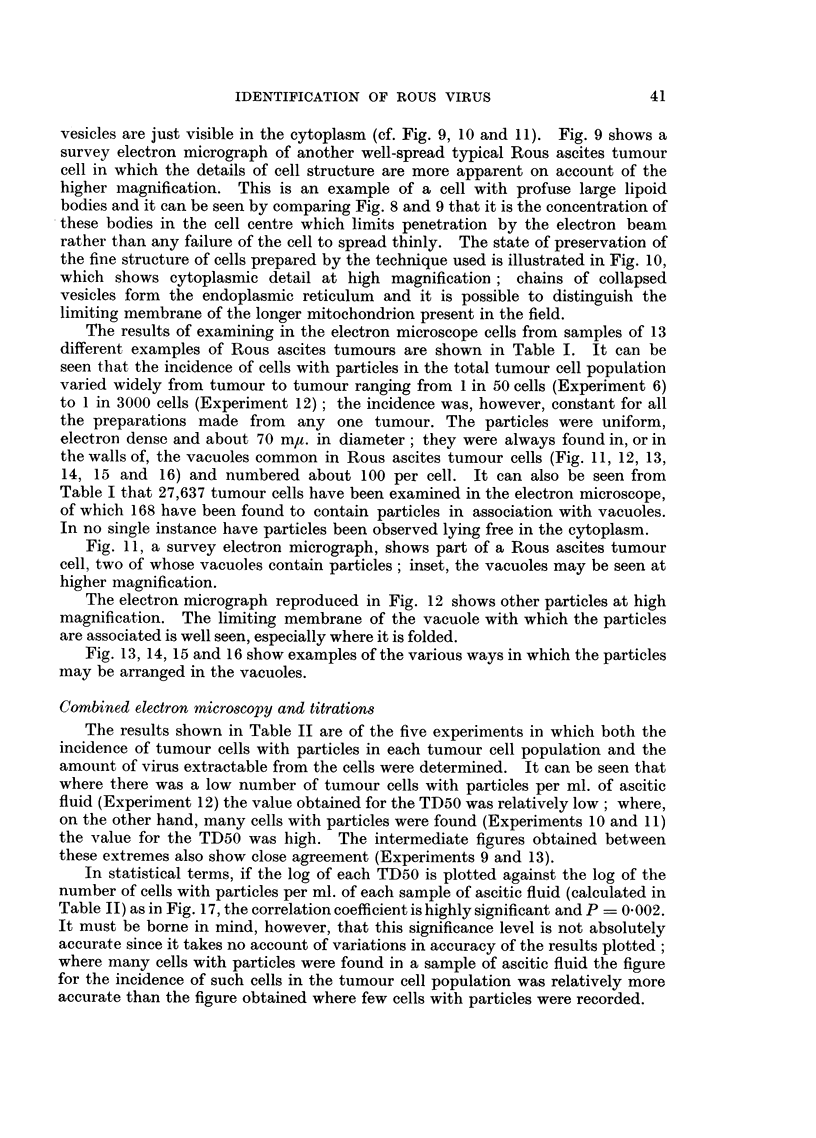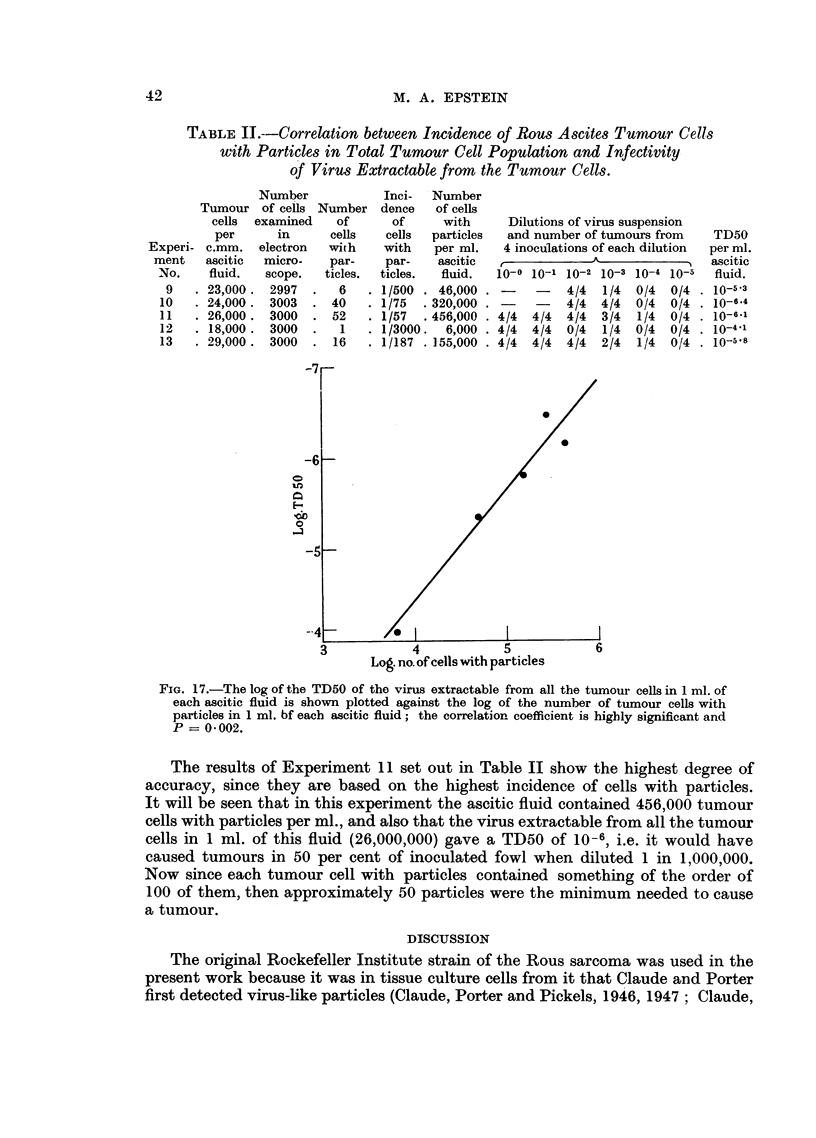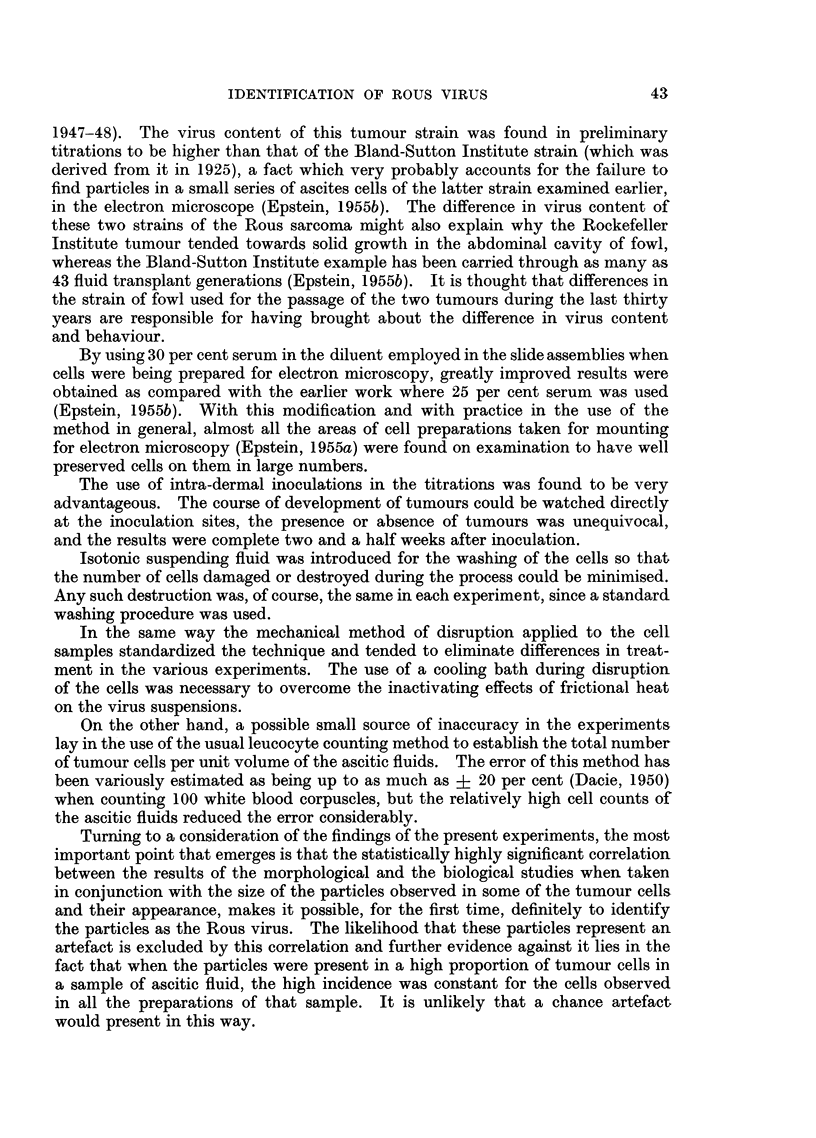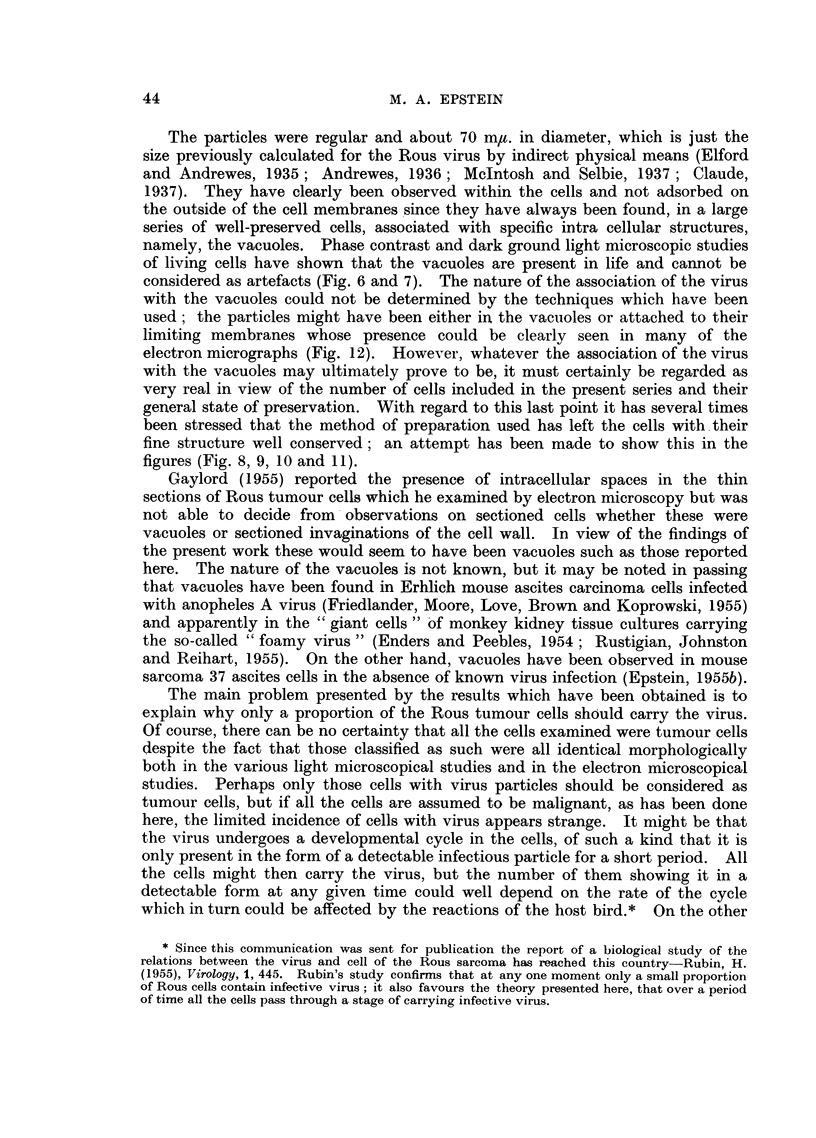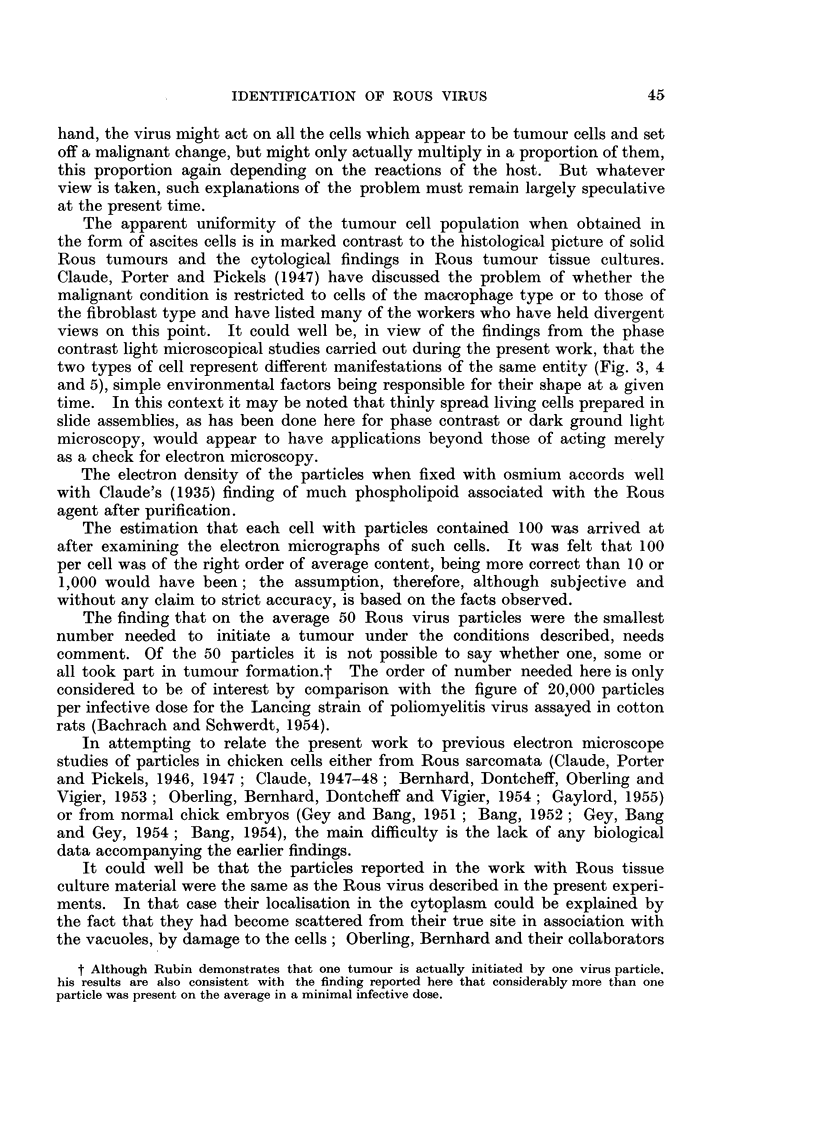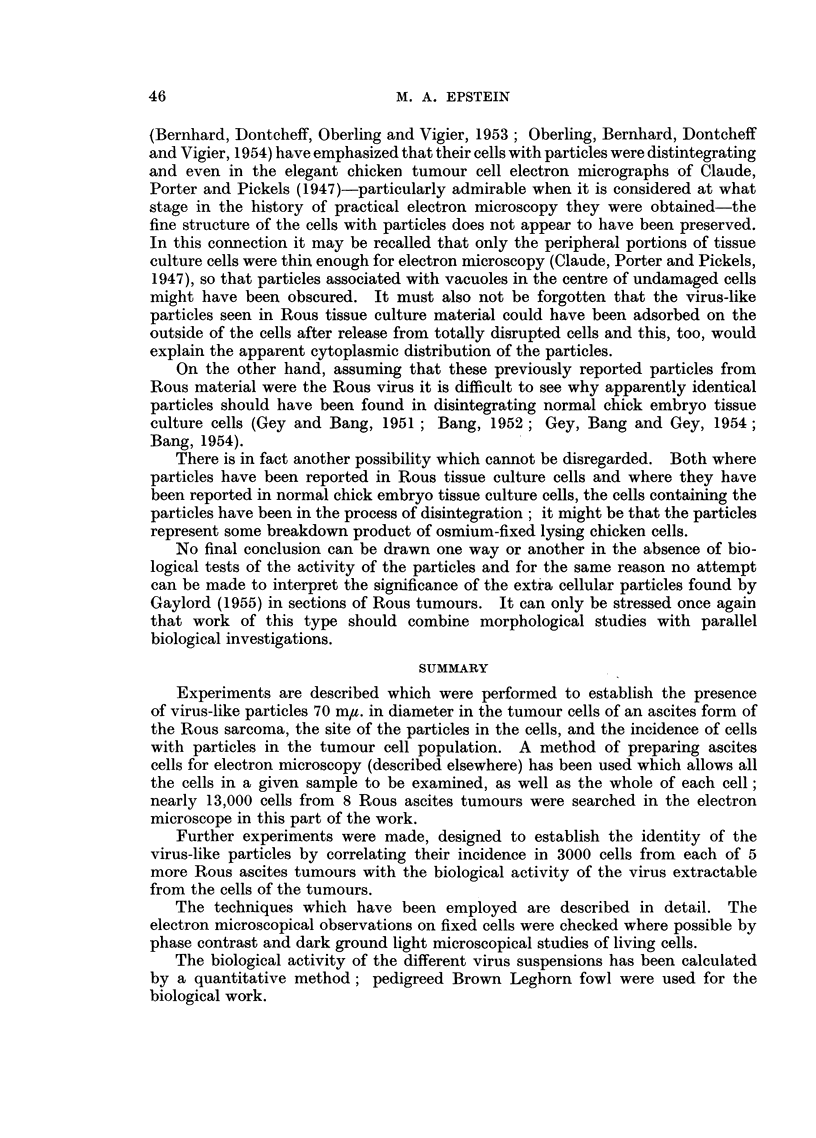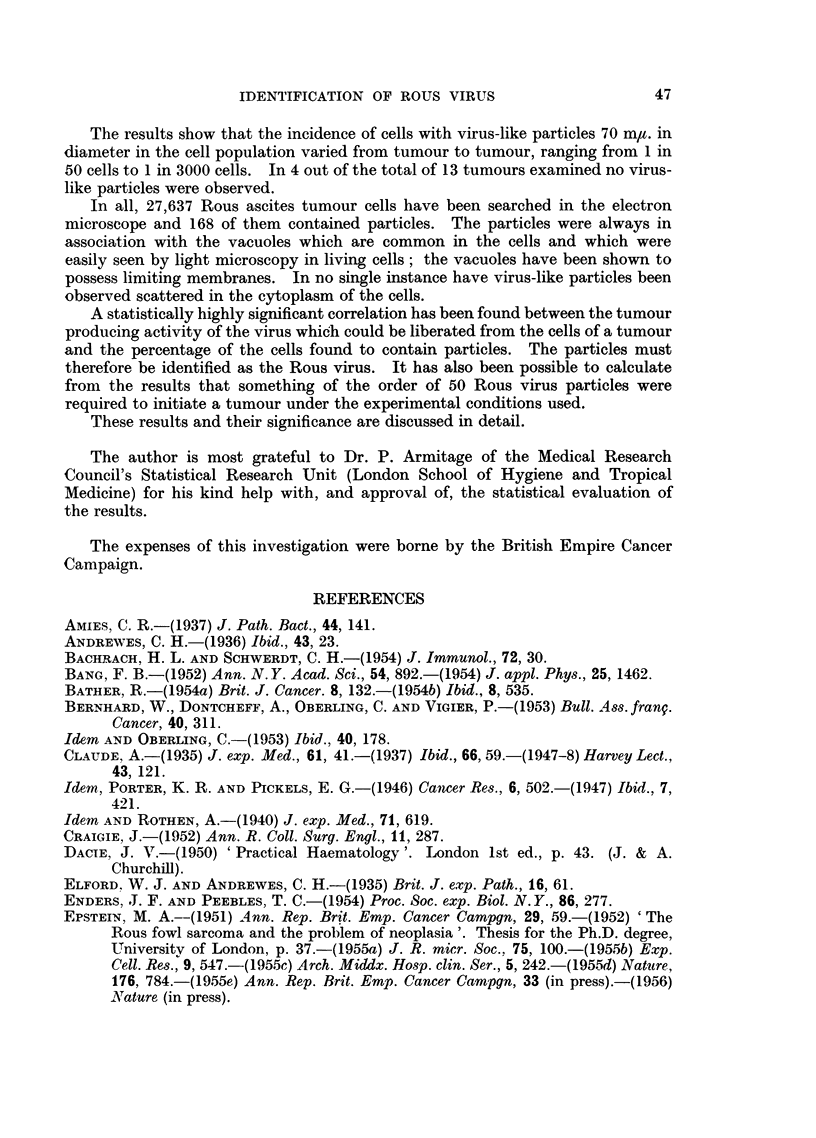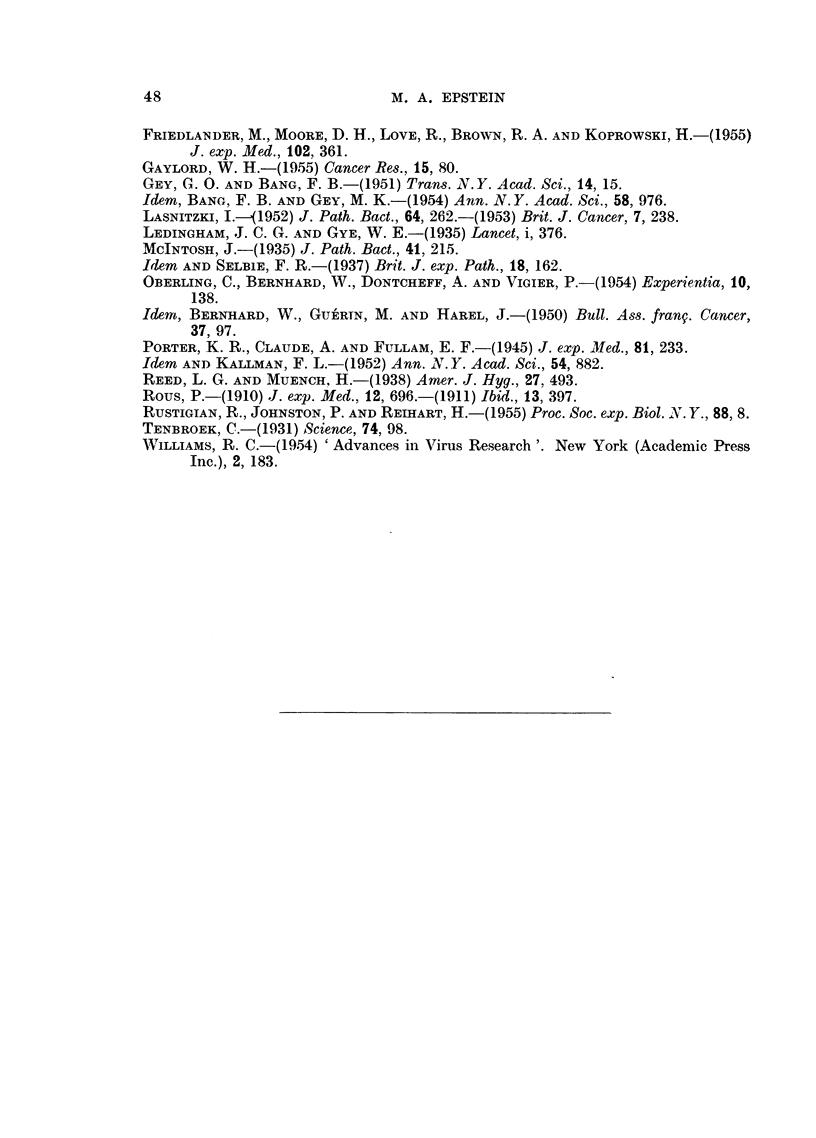# The Identification of the Rous Virus; A Morphological and Biological Study

**DOI:** 10.1038/bjc.1956.5

**Published:** 1956-03

**Authors:** M. A. Epstein

## Abstract

**Images:**


### THE IDENTIFICATION OF THE ROUS VIRUS; A MORPHOLOGICAL AND BIOLOGICAL STUDY

M. A. EPSTEIN

*From the Bland-Sutton Institute of Pathology, The Middlesex Hospital, London, W.*1

Received for publication January 31, 1956.

FORTY-FIVE years ago Rous showed that the transmissible spindle cell sarcoma of fowl which he had previously described (Rous, 1910) contained a filterable component separable from its cells and capable of infecting normal cells and making them malignant (Rous, 1911). The particulate nature of this component was demonstrated by high speed centrifugation studies 25 years later (Ledingham and Gye, 1935; McIntosh, 1935; Amies, 1937) and the same procedure was also used to estimate the size of the particles at about 70 m$\mu$. diameter (Andrewes, 1936; McIntosh and Selbie, 1937; Claude, 1937). This figure agreed well with that obtained in filtration experiments using collodion membranes of graded pore size (Elford and Andrewes, 1935).

With the first demonstration of the power of cell-free transmission of the Rous sarcoma this tumour (and the many similar malignant connective tissue tumours of fowl subsequently described) assumed great importance in the experimental pathology of cancer. The later finding that the agent responsible for cell-free transmission was a formed particle with many of the attributes of a virus, widened the field in which the Rous tumour and its agent were of interest.

Yet, in the last 12 years or so in which the general availability of the electron microscope has made it possible to examine objects of the same order of size as that established for the Rous virus by the indirect physical means described above, there have only been three reported attempts to investigate its morphology.

In the earliest work of this kind Claude and Porter examined whole Rous cells prepared for electron microscopy by their tissue culture technique (Porter, Claude and Fullam, 1945) and were able to show virus-like electron dense particles of about 75 m$\mu$. diameter scattered in the cytoplasm of some of the cells (Claude, Porter and Pickels, 1946, 1947; Claude, 1947–48). They gave no indication, however, of the frequency with which the particles were encountered, but, on the other hand, emphasised that with the method used the spread cultured cells tended to remain too thick in the central part for proper examination and the particles, in order to be seen, had to be found in the thinnest peripheral regions.

Using the method of Claude and Porter, Bernhard, Oberling and their collaborators were at first unable to confirm the presence of virus-like particles in cultured Rous cells (Oberling, Bernhard, Guérin and Harel, 1950), but it should be noted that, unlike Claude and Porter, they omitted to use in their cultures plasma from fowl known to be free from Rous antibodies; their final failure was reported at the end of five years' effort (Bernhard and Oberling, 1953). Shortly after this, however, the French workers described experiments in which virus-like particles were found with the electron microscope in Rous cells grown

*in vitro* (Bernhard, Dontcheff, Oberling and Vigier, 1953; Oberling, Bernhard, Dontcheff and Vigier, 1954). Test cultures were subjected to direct X-irradiation or were grown in media containing alcohol incorporating $C^{14}$ and 25 cells out of 6600 examined (0·38 per cent) were found to contain particles of the appropriate size; in the unirradiated control cultures 5 cells out of 3800 examined (0·13 per cent) contained similar particles. Two points are made clear in the reports of the work (Bernhard, Dontcheff, Oberling and Vigier, 1953; Oberling, Bernhard, Dontcheff and Vigier, 1954), firstly, that the plasma used in this new series of cultures was taken from young chickens to avoid the presence of Rous antibodies and secondly, that the virus-like particles were found in disintegrating cells or in cell débris.

Finally, Gaylord (1955) has made an electron microscope study of ultra-thin sections of three Rous tumours and has described particles in a predominantly extracellular situation, many in sections from one tumour, very few in those from a second tumour, and none in sections from a third.

Now it is important to note that in all the work just mentioned the material examined morphologically was never studied biologically in parallel with a view to correlating tumour-producing activity with the presence or absence of particles. Of course, in the tissue culture experiments explants were made from tumours known to have a high biological activity (Claude, Porter and Pickels, 1947; Oberling, Bernhard, Dontcheff and Vigier, 1954), but samples of the actual cells examined at the end of the period of culture were not tested for this. Indeed it is hard to see how, technically, this could have been accomplished, but even in the case of Gaylord's (1955) study of thin sections of solid tumours no biological control was attempted.

Certain virus-like particles in normal chick embryo tissue culture cells have been mentioned in passing in several reports (Gey and Bang, 1951; Bang, 1952; Gey, Bang and Gey, 1954); the particles had a diameter of 70 $m\mu$., were said to occur in distintegrating cells and to have been present in 10 per cent of cultures studied over a period of three years. In the only two micrographs published to date no recognisable cell structure is included in the field. Although this certainly might be an instance of a naturally-occurring virus being present in normal cells, the further claim which Bang (1954) has made on purely morphological grounds that this may well be the Rous virus despite its occurrence in normal chick embryos, is clearly a case in which the only evidence of value would be that of biological activity.

The difficulties of trying to equate unknown particles with virus simply on a morphological basis have recently been ably stressed by Williams (1954) and the same point has also been made by Porter and Kallman (1952). Without accompanying biological tests conclusions in work of this type founded solely on electron microscopical appearances must remain wholly speculative; where such unknown particles are observed in association with disintegrating cells the fine structure of whose other components has not been preserved, the problem becomes, as Porter and Kallman (1952) have emphasised, doubly difficult.

In view of the great uncertainty surrounding the morphology of the Rous virus it was thought that the question might best be attacked by using a method which would allow the examination in the electron microscope of all the tumour cells in a representative sample so that the incidence of cells with particles could be established. In order to eliminate the problems of sampling inherent in the examination of ultra-thin sections of Rous material for this purpose, and particularly as there was doubt as to where the particles were located in the cells, a method which also allowed the whole of each cell to be examined seemed desirable. Since, too, it was considered that parallel morphological and infectivity studies were of fundamental importance, the starting material for the work was needed in a form which could be readily handled for both these purposes. A technique fulfilling the foregoing requirements was evolved and has been applied to the tumour cells of a recently developed ascites form of the Rous sarcoma (Epstein, 1951, 1952, 1955b ; Bather, 1954*a*, 1954*b*) which, like other single dispersed tumour cells in ascitic fluid, are well suited to the taking of homogeneous representative samples.

The technique has been reported in detail elsewhere (Epstein, 1955*a*, 1955*b*). It makes use of the fact that when free round ascites tumour cells are incubated under appropriate conditions in contact with a firm surface on which they can grip, they quickly change shape from a round to a spindle form (Craigie, 1952 ; Lasnitski, 1952, 1953), an early but transient stage in this transformation consisting of the cells spreading widely and becoming thin enough for electron microscopy when the firm surface was flat.

The work described in the present communication falls into two parts. In the first part large numbers of cells from samples of eight Rous ascites tumours were examined in the electron microscope to establish the incidence of tumour cells with particles in each tumour cell population, as well as the intracellular site of the particles. In the second part the incidence of cells with particles in samples from five further Rous ascites tumours was correlated with the biological activity of the virus contained in these tumours. Brief preliminary reports have been given eleswhere of both the first series of experiments (Epstein, 1955*c* 1955*d*) and the final series (Epstein, 1955*e*, 1956).

### MATERIALS AND METHODS

*Tumour*.—The Rous No. 1 fowl sarcoma used was of the Rockefeller Institute strain derived by passage in Plymouth Rock fowl from the original tumour described by Rous (1910). It was received in July, 1954, through the kindness of Professor Albert Claude as a tumour desiccate from Rockefeller Bird No. 2931 prepared on May 27th, 1948. The tumour was passed several times by cell grafting intra-muscularly in chickens at the Bland-Sutton Institute and was then, for the present work, inoculated intra-peritoneally to convert it to an ascites form (Epstein, 1951, 1952, 1955*b* ; Bather, 1954*a* 1954*b*) which was maintained by serial passage of the fluid as described elsewhere (Epstein, 1955*b*). The ascites form of this strain of the Rous sarcoma showed an intermittent tendency to grow in solid masses in the peritoneum and 12 fluid transplant generations were the most that could be achieved. On account of this tendency to solid growth new Rous ascites tumour lines were started on several occasions in the course of the work.

*Animals*.—Pedigreed susceptible Brown Leghorn fowl from the Poultry Research Centre, Edinburgh, were used for the tumour passages and for the titrations. They were between 7 and 10½ weeks old when inoculated, with the exception of one bird aged 11½ weeks and one aged 12½. The age at which the birds were used was determined by the exigencies of supply.

*Preparation of tumour cells for electron microscopy*

The samples of Rous ascites tumour cells were prepared for electron microscopy in inverted slide assemblies by the methods previously described in detail elsewhere (Epstein, 1955*a*, 1955*b*) apart from two minor modifications. Firstly, the diluent in which the cells were placed for loading into the slide assemblies was made up to contain 30 per cent of serum from the tumour bearing fowl yielding the sample of ascitic fluid, instead of 25 per cent as in the earlier work. Secondly, although fixation was continued as before for about 22 hours in the case of at least 3 out of the 6 slide assemblies set up with each tumour cell sample, other fixation times extending up to a maximum of 30 hours were sometimes applied to the remainder.

*Techniques used in assay of virus*

When the amount of virus contained in the cells of a sample of Rous ascites fluid was to be assessed the following materials and procedures were used :

*Suspending fluid.*—The suspending fluid employed in the titrations consisted of 5 per cent inactivated rabbit serum in M/100 phosphate buffer at pH 7·38 brought to isotonicity by the addition of NaCl (0·792 g. per 100 ml.). The serum was inactivated by heating it to 56°C. for half an hour shortly before use.

*Washing of cells.*—Cells to be washed were placed in fresh suspending fluid and centrifuged in an horizontal centrifuge at 6000 r.p.m. for 5 minutes. At the end of this the suspending fluid was poured off from the deposited cells which were then considered to have been washed once ; the amount of suspending fluid used was adjusted to give 10 times the volume of the original ascitic fluid from which the cells in question had been obtained.

*Disruption of cells.*—Where cells were to be disrupted they were placed in 0·5 ml. suspending fluid and loaded into a tissue disintegrator (Ten Broek, 1931) having a ground glass piston and tube (supplied by The Ponders End Glassworks, Middlesex). The disintegrator was then attached to a 1/10 h.p. electric motor by a rubber connection joining its piston to the motor's spindle, the apparatus being so mounted as to maintain the disintegrator in an upright position. The motor was turned on and set to run so that its speed would have been 18,000 r.p.m. had the frictional resistance of the disintegrator not been present ; it was kept running for 1 minute. During this time the tube of the disintegrator was moved up and down on its rotating piston once every 10 seconds. The disintegrator was surrounded by a bath of melting ice both for 5 minutes before the motor was started and during the run.

*Preparation of virus suspensions.*—The suspension of disrupted cells was brought to 10 times the volume of the original sample of ascitic fluid from which the cells had come by the addition of more suspending fluid, except in Experiment 10, where it was made up to 20 times the volume. Allowance has been made for this difference in calculating the results. Cell débris was next deposited by centrifugation in an horizontal centrifuge at 6000 r.p.m. for 5 minutes and the resulting supernatant containing the virus liberated from the cells was decanted and kept for use as the virus suspension.

*Inoculations.*—Inoculations were made intra-dermally in the plucked breasts of fowl in a row on either side of the mid line clear of the quill-bearing areas (Fig. 1). Each inoculum consisted of 0·025 ml. and 4 fowl each received one dose of all dilutions of the virus suspension used in a given experiment. Specially sharpened intra-dermal needles were kept for the work and inoculations were made by piercing the skin and re-entering its thickness from the deep surface.

*Examination of fowl.*—The birds were examined every 2 or 3 days and all the tumours which developed (Fig. 1) were recorded. At the end of 3 weeks the birds were killed and subjected to *post mortem* examination.

### *General considerations*

Each experiment was made on a sample of ascitic fluid obtained from a different example of a Rous ascites tumour. All the samples used were from birds with recognisable abdominal swelling following intra-peritoneal inoculation of ascitic fluid 8 to 11 days previously.

The collection of samples of ascitic fluid containing the tumour cells and the counting of the cells was done by the methods which have already been reported (Epstein, 1955*b*).

The tumours were passed and the titration experiments were made with strict aseptic technique ; tests for the presence of contaminating bacteria were negative.

The time taken over the experimental procedures in the titration experiments was standardised so that in all cases the inoculations were completed about 90 minutes after killing the tumour-bearing bird.

Both the composition of the suspending fluid and the method of inoculation used in the titrations were based on techniques originally introduced by Claude (Claude and Rothen, 1940 ; Claude, 1954, personal communication).

The examination of the cells in the electron microscope was made using a screen magnification of at least 7500 $\times$ and in most cases higher magnifications as well. Only after this were the cells considered to have been searched for particles and included in the counts. Accuracy in counting was ensured by the use of a hand-type Tally Counter (of English Numbering Machines Ltd., Enfield, Middlesex).

The electron microscope employed was a Philips EM–100 of the new type 11980/23 embodying the new high resolution objective lens ; the objective aperture of $30\mu$ diameter was found to give the best image contrast. All the electron micrographs were taken using an accelerating voltage of 80 kV.

Where bright field, phase contrast, or dark ground light microscopical observations were made a Leitz Ortholux microscope was used fitted with phase contrast equipment, Heine condenser, Eisenberg warm stage and straight sided cavity slides.

If stained preparations of Rous ascites fluids were needed a wet film was prepared in the manner of a blood film, fixed when still wet in Carnoy's fluid and stained with haematoxylin and eosin.

### *Experimental procedure*

*Experiments* 1 *to* 8.—In each of these experiments Rous ascites tumour cells were prepared for electron microscopy from a sample of ascitic fluid whose tumour cell content had been counted. The preparations were then examined in the electron microscope and a record was kept both of the total number of tumour cells searched and the number of such cells found to contain virus-like particles of about 70 mμ diameter. The intracellular site of the particles was also noted.

*Experiments* 9 *to* 13.—In each of these further experiments a 1 ml. sample of ascitic fluid was divided into two portions. One portion was treated exactly as were the samples used in Experiments 1 to 8 described above. The other portion was diluted 1 in 10 in suspending fluid and the cells were collected by centrifuging in an horizontal centrifuge at 8000 r.p.m. for 10 minutes ; diluting before centrifugation reduced the viscosity of the ascitic fluid and ensured that the cells were deposited. The cells collected in this way were washed 6 times, disrupted and used to make a virus suspension which was diluted in serial tenfold steps with suspending fluid for inoculation.

### *Calculation of results*

The incidence of tumour cells with particles in the tumour cell population (Table I) was calculated from a consideration of the total number of tumour cells examined in the electrôn microscope from a sample of ascitic fluid and from the number of these cells found to have particles in them. Since, too, the total tumour cell count per c.mm. of the ascitic fluids was known, the number of tumour cells with particles per ml. of the ascitic fluids could also be worked out (Table II).

From the number of tumours which arose following the inoculations of each dilution of a virus suspension (Table II), that dilution of the virus suspension which would have caused tumours in 50 per cent of fowl when given in a dose of 1 ml., was calculated. The method of calculation used was that of Reed and Muench (1938) and the results, under the heading of TD50, have been expressed in terms of 1 ml. of the original samples of ascitic fluid containing the cells from which the virus suspensions were made (Table II).

### RESULTS

### *Light microscopy*

Throughout the work Rous ascites fluids have been found to contain about 20,000 free round tumour cells per c.mm. amongst which mitoses were frequently seen. In addition a variable number of erythrocytes and a relatively small number of white blood corpuscles were present. The tumour cells were sometimes encountered in small clumps, but whether seen thus or dispersed singly, their morphology was the same. These findings are based on observations made on ascitic fluids diluted and placed in a haemocytometer for counting, on phase contrast and dark ground studies of the living cells of ascitic fluids in slide assemblies and on examinations of fixed, stained, smears of ascites cells. A photo micrograph of a typical stained smear is shown in Fig. 2. Many large round single ascites tumour cells are present, one showing a characteristic mitosis ; in addition the nuclei of erythrocytes lysed by the fixative can be seen, together with a few white blood corpuscles.

Phase contrast studies of living Rous ascites cells in slide assemblies have confirmed that the spreading of the cells is a transient stage in the change of shape which they undergo from round to spindle form when in contact with a firm flat surface on which they can grip, rather than a function of the motility of the cells (Epstein, 1955*b*). Fig. 3 shows a phase contrast photo micrograph of the floor of an inverted slide assembly after 3 minutes incubation at 37° C ; many of the free round ascites cells have come to rest on the floor, whilst others, surrounded by a diffraction halo, have not yet reached the floor being just above this level and out of the plane of exact focus. Yet other cells, still sinking through the diluent well above the plane of focus, are responsible for causing the diffraction rings. Fig. 4, also a phase contrast photo micrograph, shows the floor of a slide assembly after 15 minutes incubation at 37° C ; it can be seen that all the cells have now sunk to the floor and that they have either spread widely or are in the process of doing so. In the phase contrast photo micrograph shown in Fig. 5, many of the live Rous ascites tumour cells on the floor of a slide assembly have, after 2 hours incubation at 37°C., already completed the change from round to spindle form. Others can be seen starting this transformation which was usually complete after about 4 hours incubation.

High power phase contrast and dark ground observations on living Rous ascites tumour cells have been made for comparison with electron micrographs of the cells after osmium fixation. The phase contrast photo micrograph shown in Fig. 6 is of a well-spread tumour cell with prominent nucleus and nucleolus, many small lipoid bodies, vacuoles of various sizes and a number of filamentous mitochondria. In Fig. 7 a dark ground photo micrograph of an exactly similar cell is shown ; the lipoid bodies stand out with great brilliance and the shimmering appearance of the vacuoles is also apparent.

### *Electron microscopy*

All the structures which could be seen by phase contrast and dark ground microscopy in living Rous ascites tumour cells were present in the osmium-fixed cells viewed in the electron microscope. Fig. 8 shows an electron micrograph of such a fixed cell reproduced at the same magnification as the exactly similar living cells shown in Fig. 6 and 7 ; the only additional structure evident in the electron micrograph is the endoplasmic reticulum whose chains of collapsed

TABLE I.—*Incidence of Rous Ascites Tumour Cells with Particles in Total Tumour Cell Population of Various Examples of Rous Ascites Tumours, and Intracellular Site of Particles.*

| Experiment No. | Number of cells examined in electron microscope. | Number of cells with particles. | Incidence of cells with particles. | Site of particles | |
|---|---|---|---|---|---|
| | | | | Vacuole. | Elsewhere. |
| 1 | 150 | 1 | ? | 1 | 0 |
| 2 | 2030 | 0 | — | — | — |
| 3 | 1758 | 0 | — | — | — |
| 4 | 2114 | 4 | 1/500 | 4 | 0 |
| 5 | 1202 | 0 | — | — | — |
| 6 | 2357 | 47 | 1/50 | 47 | 0 |
| 7 | 2000 | 1 | 1/2000 | 1 | 0 |
| 8 | 1026 | 0 | — | — | — |
| 9 | 2997 | 6 | 1/500 | 6 | 0 |
| 10 | 3003 | 40 | 1/75 | 40 | 0 |
| 11 | 3000 | 52 | 1/57 | 52 | 0 |
| 12 | 3000 | 1 | 1/3000 | 1 | 0 |
| 13 | 3000 | 16 | 1/187 | 16 | 0 |
| Totals | 27,637 | 168 | — | 168 | 0 |

### EXPLANATION OF PLATES

FIG. 1.—Left (*a*) and right (*b*) sides of the plucked breast of a chicken in which intra-dermal inoculations of a series of 6 serial dilutions of a Rous virus suspension had been made 11 days previously. Tumours have developed from the first four dilutions and in the case of the three most potent of these the haemorrhages which have occurred in the tumours and the nodules at the points where the needles pierced the skin, can be seen. × 2.

FIG. 2.—Smear of Rous ascitic fluid. Numerous large tumour cells are present, one in mitosis ; the nuclei of erythrocytes lysed during fixation can be seen as well as 4 or 5 white blood cells. Carnoy's fixation when wet ; haematoxylin and eosin. Photo micrograph . × 650.

FIG. 3.—Floor of an inverted slide assembly after 3 minutes incubation at 37° C. showing many of the living round Rous ascites tumour cells at rest. Other cells still sinking through the diluent above the plane of focus are surrounded by diffraction haloes, or at higher levels, are responsible for causing the diffraction rings. Phase contrast photo micrograph. × 190.

FIG. 4.—Floor of an inverted slide assembly after 15 minutes incubation at 37° C. All the cells have now sunk down and have either spread widely or are in the process of doing so. Phase contrast photo micrograph. × 190.

FIG. 5.—Floor of an inverted slide assembly after 2 hours incubation at 37° C. Many of the live Rous ascites tumour cells have already completed the change from round to spindle form ; other are starting this transformation. Phase contrast photo micrograph. × 190.

FIG. 6.—Well spread living Rous ascites tumour cell having on the left of its centre a prominent nucleus with a nucleolus ; many small dark lipoid bodies, clear vacuoles of various sizes, and a number of filamentous mitochondria can also be seen. Phase contrast photo micrograph. × 1,500.

FIG. 7.—Well spread living Rous ascites tumour cell ; the lipoid bodies stand out with great brilliance, and the shimmering appearance of three vacuoles is apparent. The nucleus occupies the kidney shaped space on the left of the cell's centre and can be seen to contain a nucleolus. Dark ground photo micrograph. × 1,500.

FIG. 8.—Well spread osmium fixed Rous ascites tumour cell showing all the features present in the living cell shown in Fig. 6. In addition the chains of collapsed vesicles of the endoplasmic reticulum are just visible in the cytoplasm. Electron micrograph. × 1,500.

FIG. 9.—Survey picture of a well spread typical Rous ascites tumour cell. The nucleus (N) is surrounded on three sides by profuse large osmiophilic lipoid bodies (L) ; the cytoplasm contains the chains of collapsed vesicles of the endoplasmic reticulum (R) as well as many filamentous mitochondria (M). Numerous vacuoles of various sizes (V) are also present (cf. Fig. 6, 7 and 8). This is an example of a cell with profuse large lipoid bodies which tend to limit observation in the cell centre. Electron micrograph ; osmium vapour fixation for 30 hours. × 3,000.

FIG. 10.—Cytoplasmic detail of a Rous ascites tumour cell. The chains of collapsed vesicles of the endoplasmic reticulum can be seen, as well as the limiting membrane of the longer mitochondrion. Electron micrograph ; osmium vapour fixation for 30 hours. × 20,000.

FIG. 11.—Survey picture of part of a Rous ascites tumour cell with two vacuoles containing virus particles ; the nucleus, mitochondria and endoplasmic reticulum are well preserved. Electron micrograph ; osmium vapour fixation for 30 hours. × 6,000. Inset the vacuoles may be seen at higher magnification. × 12,000.

FIG. 12.—Large vacuole in a Rous ascites tumour cell showing the Rous virus particles. The limiting membrane of the vacuole can be distinguished particularly where it has become folded. Two smaller vacuoles occupy the top of the field and their membranes are also evident. Electron micrograph ; osmium vapour fixation for 30 hours. × 25,000.

FIG. 13, 14, 15 and 16.—Small vacuoles from Rous ascites tumour cells showing the different ways in which the virus particles may be arranged in the vacuoles. Folds in the limiting membranes of the vacuoles can be seen in Fig. 14 and 16, which also show portions of mitochondria, as does Fig. 13. Electron micrographs ; osmium vapour fixation for 30 hours. × 20,000.

1a
1b

10μ
2

Epstein.

L
V
R
N
M
1μ
9

1μ
10

Epstein.

Epstein.

Epstein.

vesicles are just visible in the cytoplasm (cf. Fig. 9, 10 and 11). Fig. 9 shows a survey electron micrograph of another well-spread typical Rous ascites tumour cell in which the details of cell structure are more apparent on account of the higher magnification. This is an example of a cell with profuse large lipoid bodies and it can be seen by comparing Fig. 8 and 9 that it is the concentration of these bodies in the cell centre which limits penetration by the electron beam rather than any failure of the cell to spread thinly. The state of preservation of the fine structure of cells prepared by the technique used is illustrated in Fig. 10, which shows cytoplasmic detail at high magnification ; chains of collapsed vesicles form the endoplasmic reticulum and it is possible to distinguish the limiting membrane of the longer mitochondrion present in the field.

The results of examining in the electron microscope cells from samples of 13 different examples of Rous ascites tumours are shown in Table I. It can be seen that the incidence of cells with particles in the total tumour cell population varied widely from tumour to tumour ranging from 1 in 50 cells (Experiment 6) to 1 in 3000 cells (Experiment 12) ; the incidence was, however, constant for all the preparations made from any one tumour. The particles were uniform, electron dense and about 70 m$\mu$. in diameter ; they were always found in, or in the walls of, the vacuoles common in Rous ascites tumour cells (Fig. 11, 12, 13, 14, 15 and 16) and numbered about 100 per cell. It can also be seen from Table I that 27,637 tumour cells have been examined in the electron microscope, of which 168 have been found to contain particles in association with vacuoles. In no single instance have particles been observed lying free in the cytoplasm.

Fig. 11, a survey electron micrograph, shows part of a Rous ascites tumour cell, two of whose vacuoles contain particles ; inset, the vacuoles may be seen at higher magnification.

The electron micrograph reproduced in Fig. 12 shows other particles at high magnification. The limiting membrane of the vacuole with which the particles are associated is well seen, especially where it is folded.

Fig. 13, 14, 15 and 16 show examples of the various ways in which the particles may be arranged in the vacuoles.

*Combined electron microscopy and titrations*

The results shown in Table II are of the five experiments in which both the incidence of tumour cells with particles in each tumour cell population and the amount of virus extractable from the cells were determined. It can be seen that where there was a low number of tumour cells with particles per ml. of ascitic fluid (Experiment 12) the value obtained for the TD50 was relatively low ; where, on the other hand, many cells with particles were found (Experiments 10 and 11) the value for the TD50 was high. The intermediate figures obtained between these extremes also show close agreement (Experiments 9 and 13).

In statistical terms, if the log of each TD50 is plotted against the log of the number of cells with particles per ml. of each sample of ascitic fluid (calculated in Table II) as in Fig. 17, the correlation coefficient is highly significant and $P = 0{\cdot}002$. It must be borne in mind, however, that this significance level is not absolutely accurate since it takes no account of variations in accuracy of the results plotted ; where many cells with particles were found in a sample of ascitic fluid the figure for the incidence of such cells in the tumour cell population was relatively more accurate than the figure obtained where few cells with particles were recorded.

TABLE II.—*Correlation between Incidence of Rous Ascites Tumour Cells with Particles in Total Tumour Cell Population and Infectivity of Virus Extractable from the Tumour Cells.*

| Experiment No. | Tumour cells per c.mm. ascitic fluid. | Number of cells examined in electron microscope. | Number of cells with particles. | Incidence of cells with particles. | Number of cells with particles per ml. ascitic fluid. | Dilutions of virus suspension and number of tumours from 4 inoculations of each dilution: $10^{-0}$ | $10^{-1}$ | $10^{-2}$ | $10^{-3}$ | $10^{-4}$ | $10^{-5}$ | TD50 per ml. ascitic fluid. |
|---|---|---|---|---|---|---|---|---|---|---|---|---|
| 9 | 23,000 | 2997 | 6 | 1/500 | 46,000 | — | — | 4/4 | 1/4 | 0/4 | 0/4 | $10^{-5 \cdot 3}$ |
| 10 | 24,000 | 3003 | 40 | 1/75 | 320,000 | — | — | 4/4 | 4/4 | 0/4 | 0/4 | $10^{-6 \cdot 4}$ |
| 11 | 26,000 | 3000 | 52 | 1/57 | 456,000 | 4/4 | 4/4 | 4/4 | 3/4 | 1/4 | 0/4 | $10^{-6 \cdot 1}$ |
| 12 | 18,000 | 3000 | 1 | 1/3000 | 6,000 | 4/4 | 4/4 | 0/4 | 1/4 | 0/4 | 0/4 | $10^{-4 \cdot 1}$ |
| 13 | 29,000 | 3000 | 16 | 1/187 | 155,000 | 4/4 | 4/4 | 4/4 | 2/4 | 1/4 | 0/4 | $10^{-5 \cdot 8}$ |

FIG. 17.—The log of the TD50 of the virus extractable from all the tumour cells in 1 ml. of each ascitic fluid is shown plotted against the log of the number of tumour cells with particles in 1 ml. of each ascitic fluid; the correlation coefficient is highly significant and $P = 0 \cdot 002$.

The results of Experiment 11 set out in Table II show the highest degree of accuracy, since they are based on the highest incidence of cells with particles. It will be seen that in this experiment the ascitic fluid contained 456,000 tumour cells with particles per ml., and also that the virus extractable from all the tumour cells in 1 ml. of this fluid (26,000,000) gave a TD50 of $10^{-6}$, i.e. it would have caused tumours in 50 per cent of inoculated fowl when diluted 1 in 1,000,000. Now since each tumour cell with particles contained something of the order of 100 of them, then approximately 50 particles were the minimum needed to cause a tumour.

DISCUSSION

The original Rockefeller Institute strain of the Rous sarcoma was used in the present work because it was in tissue culture cells from it that Claude and Porter first detected virus-like particles (Claude, Porter and Pickels, 1946, 1947 ; Claude,

1947–48). The virus content of this tumour strain was found in preliminary titrations to be higher than that of the Bland-Sutton Institute strain (which was derived from it in 1925), a fact which very probably accounts for the failure to find particles in a small series of ascites cells of the latter strain examined earlier, in the electron microscope (Epstein, 1955*b*). The difference in virus content of these two strains of the Rous sarcoma might also explain why the Rockefeller Institute tumour tended towards solid growth in the abdominal cavity of fowl, whereas the Bland-Sutton Institute example has been carried through as many as 43 fluid transplant generations (Epstein, 1955*b*). It is thought that differences in the strain of fowl used for the passage of the two tumours during the last thirty years are responsible for having brought about the difference in virus content and behaviour.

By using 30 per cent serum in the diluent employed in the slide assemblies when cells were being prepared for electron microscopy, greatly improved results were obtained as compared with the earlier work where 25 per cent serum was used (Epstein, 1955*b*). With this modification and with practice in the use of the method in general, almost all the areas of cell preparations taken for mounting for electron microscopy (Epstein, 1955*a*) were found on examination to have well preserved cells on them in large numbers.

The use of intra-dermal inoculations in the titrations was found to be very advantageous. The course of development of tumours could be watched directly at the inoculation sites, the presence or absence of tumours was unequivocal, and the results were complete two and a half weeks after inoculation.

Isotonic suspending fluid was introduced for the washing of the cells so that the number of cells damaged or destroyed during the process could be minimised. Any such destruction was, of course, the same in each experiment, since a standard washing procedure was used.

In the same way the mechanical method of disruption applied to the cell samples standardized the technique and tended to eliminate differences in treatment in the various experiments. The use of a cooling bath during disruption of the cells was necessary to overcome the inactivating effects of frictional heat on the virus suspensions.

On the other hand, a possible small source of inaccuracy in the experiments lay in the use of the usual leucocyte counting method to establish the total number of tumour cells per unit volume of the ascitic fluids. The error of this method has been variously estimated as being up to as much as $\pm$ 20 per cent (Dacie, 1950) when counting 100 white blood corpuscles, but the relatively high cell counts of the ascitic fluids reduced the error considerably.

Turning to a consideration of the findings of the present experiments, the most important point that emerges is that the statistically highly significant correlation between the results of the morphological and the biological studies when taken in conjunction with the size of the particles observed in some of the tumour cells and their appearance, makes it possible, for the first time, definitely to identify the particles as the Rous virus. The likelihood that these particles represent an artefact is excluded by this correlation and further evidence against it lies in the fact that when the particles were present in a high proportion of tumour cells in a sample of ascitic fluid, the high incidence was constant for the cells observed in all the preparations of that sample. It is unlikely that a chance artefact would present in this way.

The particles were regular and about 70 mμ. in diameter, which is just the size previously calculated for the Rous virus by indirect physical means (Elford and Andrewes, 1935; Andrewes, 1936; McIntosh and Selbie, 1937; Claude, 1937). They have clearly been observed within the cells and not adsorbed on the outside of the cell membranes since they have always been found, in a large series of well-preserved cells, associated with specific intra cellular structures, namely, the vacuoles. Phase contrast and dark ground light microscopic studies of living cells have shown that the vacuoles are present in life and cannot be considered as artefacts (Fig. 6 and 7). The nature of the association of the virus with the vacuoles could not be determined by the techniques which have been used; the particles might have been either in the vacuoles or attached to their limiting membranes whose presence could be clearly seen in many of the electron micrographs (Fig. 12). However, whatever the association of the virus with the vacuoles may ultimately prove to be, it must certainly be regarded as very real in view of the number of cells included in the present series and their general state of preservation. With regard to this last point it has several times been stressed that the method of preparation used has left the cells with their fine structure well conserved; an attempt has been made to show this in the figures (Fig. 8, 9, 10 and 11).

Gaylord (1955) reported the presence of intracellular spaces in the thin sections of Rous tumour cells which he examined by electron microscopy but was not able to decide from observations on sectioned cells whether these were vacuoles or sectioned invaginations of the cell wall. In view of the findings of the present work these would seem to have been vacuoles such as those reported here. The nature of the vacuoles is not known, but it may be noted in passing that vacuoles have been found in Erhlich mouse ascites carcinoma cells infected with anopheles A virus (Friedlander, Moore, Love, Brown and Koprowski, 1955) and apparently in the "giant cells" of monkey kidney tissue cultures carrying the so-called "foamy virus" (Enders and Peebles, 1954; Rustigian, Johnston and Reihart, 1955). On the other hand, vacuoles have been observed in mouse sarcoma 37 ascites cells in the absence of known virus infection (Epstein, 1955*b*).

The main problem presented by the results which have been obtained is to explain why only a proportion of the Rous tumour cells should carry the virus. Of course, there can be no certainty that all the cells examined were tumour cells despite the fact that those classified as such were all identical morphologically both in the various light microscopical studies and in the electron microscopical studies. Perhaps only those cells with virus particles should be considered as tumour cells, but if all the cells are assumed to be malignant, as has been done here, the limited incidence of cells with virus appears strange. It might be that the virus undergoes a developmental cycle in the cells, of such a kind that it is only present in the form of a detectable infectious particle for a short period. All the cells might then carry the virus, but the number of them showing it in a detectable form at any given time could well depend on the rate of the cycle which in turn could be affected by the reactions of the host bird.* On the other

* Since this communication was sent for publication the report of a biological study of the relations between the virus and cell of the Rous sarcoma has reached this country—Rubin, H. (1955), *Virology*, **1**, 445. Rubin's study confirms that at any one moment only a small proportion of Rous cells contain infective virus; it also favours the theory presented here, that over a period of time all the cells pass through a stage of carrying infective virus.

hand, the virus might act on all the cells which appear to be tumour cells and set off a malignant change, but might only actually multiply in a proportion of them, this proportion again depending on the reactions of the host. But whatever view is taken, such explanations of the problem must remain largely speculative at the present time.

The apparent uniformity of the tumour cell population when obtained in the form of ascites cells is in marked contrast to the histological picture of solid Rous tumours and the cytological findings in Rous tumour tissue cultures. Claude, Porter and Pickels (1947) have discussed the problem of whether the malignant condition is restricted to cells of the macrophage type or to those of the fibroblast type and have listed many of the workers who have held divergent views on this point. It could well be, in view of the findings from the phase contrast light microscopical studies carried out during the present work, that the two types of cell represent different manifestations of the same entity (Fig. 3, 4 and 5), simple environmental factors being responsible for their shape at a given time. In this context it may be noted that thinly spread living cells prepared in slide assemblies, as has been done here for phase contrast or dark ground light microscopy, would appear to have applications beyond those of acting merely as a check for electron microscopy.

The electron density of the particles when fixed with osmium accords well with Claude's (1935) finding of much phospholipoid associated with the Rous agent after purification.

The estimation that each cell with particles contained 100 was arrived at after examining the electron micrographs of such cells. It was felt that 100 per cell was of the right order of average content, being more correct than 10 or 1,000 would have been; the assumption, therefore, although subjective and without any claim to strict accuracy, is based on the facts observed.

The finding that on the average 50 Rous virus particles were the smallest number needed to initiate a tumour under the conditions described, needs comment. Of the 50 particles it is not possible to say whether one, some or all took part in tumour formation.† The order of number needed here is only considered to be of interest by comparison with the figure of 20,000 particles per infective dose for the Lancing strain of poliomyelitis virus assayed in cotton rats (Bachrach and Schwerdt, 1954).

In attempting to relate the present work to previous electron microscope studies of particles in chicken cells either from Rous sarcomata (Claude, Porter and Pickels, 1946, 1947; Claude, 1947–48; Bernhard, Dontcheff, Oberling and Vigier, 1953; Oberling, Bernhard, Dontcheff and Vigier, 1954; Gaylord, 1955) or from normal chick embryos (Gey and Bang, 1951; Bang, 1952; Gey, Bang and Gey, 1954; Bang, 1954), the main difficulty is the lack of any biological data accompanying the earlier findings.

It could well be that the particles reported in the work with Rous tissue culture material were the same as the Rous virus described in the present experiments. In that case their localisation in the cytoplasm could be explained by the fact that they had become scattered from their true site in association with the vacuoles, by damage to the cells; Oberling, Bernhard and their collaborators

† Although Rubin demonstrates that one tumour is actually initiated by one virus particle, his results are also consistent with the finding reported here that considerably more than one particle was present on the average in a minimal infective dose.

(Bernhard, Dontcheff, Oberling and Vigier, 1953 ; Oberling, Bernhard, Dontcheff and Vigier, 1954) have emphasized that their cells with particles were distintegrating and even in the elegant chicken tumour cell electron micrographs of Claude, Porter and Pickels (1947)—particularly admirable when it is considered at what stage in the history of practical electron microscopy they were obtained—the fine structure of the cells with particles does not appear to have been preserved. In this connection it may be recalled that only the peripheral portions of tissue culture cells were thin enough for electron microscopy (Claude, Porter and Pickels, 1947), so that particles associated with vacuoles in the centre of undamaged cells might have been obscured. It must also not be forgotten that the virus-like particles seen in Rous tissue culture material could have been adsorbed on the outside of the cells after release from totally disrupted cells and this, too, would explain the apparent cytoplasmic distribution of the particles.

On the other hand, assuming that these previously reported particles from Rous material were the Rous virus it is difficult to see why apparently identical particles should have been found in disintegrating normal chick embryo tissue culture cells (Gey and Bang, 1951 ; Bang, 1952 ; Gey, Bang and Gey, 1954 ; Bang, 1954).

There is in fact another possibility which cannot be disregarded. Both where particles have been reported in Rous tissue culture cells and where they have been reported in normal chick embryo tissue culture cells, the cells containing the particles have been in the process of disintegration ; it might be that the particles represent some breakdown product of osmium-fixed lysing chicken cells.

No final conclusion can be drawn one way or another in the absence of biological tests of the activity of the particles and for the same reason no attempt can be made to interpret the significance of the extra cellular particles found by Gaylord (1955) in sections of Rous tumours. It can only be stressed once again that work of this type should combine morphological studies with parallel biological investigations.

SUMMARY

Experiments are described which were performed to establish the presence of virus-like particles 70 m$\mu$. in diameter in the tumour cells of an ascites form of the Rous sarcoma, the site of the particles in the cells, and the incidence of cells with particles in the tumour cell population. A method of preparing ascites cells for electron microscopy (described elsewhere) has been used which allows all the cells in a given sample to be examined, as well as the whole of each cell ; nearly 13,000 cells from 8 Rous ascites tumours were searched in the electron microscope in this part of the work.

Further experiments were made, designed to establish the identity of the virus-like particles by correlating their incidence in 3000 cells from each of 5 more Rous ascites tumours with the biological activity of the virus extractable from the cells of the tumours.

The techniques which have been employed are described in detail. The electron microscopical observations on fixed cells were checked where possible by phase contrast and dark ground light microscopical studies of living cells.

The biological activity of the different virus suspensions has been calculated by a quantitative method ; pedigreed Brown Leghorn fowl were used for the biological work.

The results show that the incidence of cells with virus-like particles 70 m$\mu$. in diameter in the cell population varied from tumour to tumour, ranging from 1 in 50 cells to 1 in 3000 cells. In 4 out of the total of 13 tumours examined no virus-like particles were observed.

In all, 27,637 Rous ascites tumour cells have been searched in the electron microscope and 168 of them contained particles. The particles were always in association with the vacuoles which are common in the cells and which were easily seen by light microscopy in living cells ; the vacuoles have been shown to possess limiting membranes. In no single instance have virus-like particles been observed scattered in the cytoplasm of the cells.

A statistically highly significant correlation has been found between the tumour producing activity of the virus which could be liberated from the cells of a tumour and the percentage of the cells found to contain particles. The particles must therefore be identified as the Rous virus. It has also been possible to calculate from the results that something of the order of 50 Rous virus particles were required to initiate a tumour under the experimental conditions used.

These results and their significance are discussed in detail.

The author is most grateful to Dr. P. Armitage of the Medical Research Council's Statistical Research Unit (London School of Hygiene and Tropical Medicine) for his kind help with, and approval of, the statistical evaluation of the results.

The expenses of this investigation were borne by the British Empire Cancer Campaign.

### REFERENCES

Amies, C. R.—(1937) *J. Path. Bact.*, **44**, 141.
Andrewes, C. H.—(1936) *Ibid.*, **43**, 23.
Bachrach, H. L. and Schwerdt, C. H.—(1954) *J. Immunol.*, **72**, 30.
Bang, F. B.—(1952) *Ann. N.Y. Acad. Sci.*, **54**, 892.—(1954) *J. appl. Phys.*, **25**, 1462.
Bather, R.—(1954*a*) *Brit. J. Cancer.* **8**, 132.—(1954*b*) *Ibid.*, **8**, 535.
Bernhard, W., Dontcheff, A., Oberling, C. and Vigier, P.—(1953) *Bull. Ass. franç. Cancer*, **40**, 311.
*Idem* and Oberling, C.—(1953) *Ibid.*, **40**, 178.
Claude, A.—(1935) *J. exp. Med.*, **61**, 41.—(1937) *Ibid.*, **66**, 59.—(1947–8) *Harvey Lect.*, **43**, 121.
*Idem*, Porter, K. R. and Pickels, E. G.—(1946) *Cancer Res.*, **6**, 502.—(1947) *Ibid.*, **7**, 421.
*Idem* and Rothen, A.—(1940) *J. exp. Med.*, **71**, 619.
Craigie, J.—(1952) *Ann. R. Coll. Surg. Engl.*, **11**, 287.
Dacie, J. V.—(1950) 'Practical Haematology'. London 1st ed., p. 43. (J. & A. Churchill).
Elford, W. J. and Andrewes, C. H.—(1935) *Brit. J. exp. Path.*, **16**, 61.
Enders, J. F. and Peebles, T. C.—(1954) *Proc. Soc. exp. Biol. N.Y.*, **86**, 277.
Epstein, M. A.—(1951) *Ann. Rep. Brit. Emp. Cancer Campgn*, **29**, 59.—(1952) 'The Rous fowl sarcoma and the problem of neoplasia'. Thesis for the Ph.D. degree, University of London, p. 37.—(1955*a*) *J. R. micr. Soc.*, **75**, 100.—(1955*b*) *Exp. Cell. Res.*, **9**, 547.—(1955*c*) *Arch. Middx. Hosp. clin. Ser.*, **5**, 242.—(1955*d*) *Nature*, **176**, 784.—(1955*e*) *Ann. Rep. Brit. Emp. Cancer Campgn*, **33** (in press).—(1956) *Nature* (in press).

Friedlander, M., Moore, D. H., Love, R., Brown, R. A. and Koprowski, H.—(1955) *J. exp. Med.*, **102**, 361.
Gaylord, W. H.—(1955) *Cancer Res.*, **15**, 80.
Gey, G. O. and Bang, F. B.—(1951) *Trans. N.Y. Acad. Sci.*, **14**, 15.
*Idem*, Bang, F. B. and Gey, M. K.—(1954) *Ann. N.Y. Acad. Sci.*, **58**, 976.
Lasnitzki, I.—(1952) *J. Path. Bact.*, **64**, 262.—(1953) *Brit. J. Cancer*, **7**, 238.
Ledingham, J. C. G. and Gye, W. E.—(1935) *Lancet*, i, 376.
McIntosh, J.—(1935) *J. Path. Bact.*, **41**, 215.
*Idem* and Selbie, F. R.—(1937) *Brit. J. exp. Path.*, **18**, 162.
Oberling, C., Bernhard, W., Dontcheff, A. and Vigier, P.—(1954) *Experientia*, **10**, 138.
*Idem*, Bernhard, W., Guérin, M. and Harel, J.—(1950) *Bull. Ass. franç. Cancer*, **37**, 97.
Porter, K. R., Claude, A. and Fullam, E. F.—(1945) *J. exp. Med.*, **81**, 233.
*Idem* and Kallman, F. L.—(1952) *Ann. N.Y. Acad. Sci.*, **54**, 882.
Reed, L. G. and Muench, H.—(1938) *Amer. J. Hyg.*, **27**, 493.
Rous, P.—(1910) *J. exp. Med.*, **12**, 696.—(1911) *Ibid.*, **13**, 397.
Rustigian, R., Johnston, P. and Reihart, H.—(1955) *Proc. Soc. exp. Biol. N.Y.*, **88**, 8.
Tenbroek, C.—(1931) *Science*, **74**, 98.
Williams, R. C.—(1954) ‘ Advances in Virus Research ’. New York (Academic Press Inc.), **2**, 183.